# Artificial intelligence driven design of catalysts and materials for ring opening polymerization using a domain-specific language

**DOI:** 10.1038/s41467-023-39396-3

**Published:** 2023-06-21

**Authors:** Nathaniel H. Park, Matteo Manica, Jannis Born, James L. Hedrick, Tim Erdmann, Dmitry Yu. Zubarev, Nil Adell-Mill, Pedro L. Arrechea

**Affiliations:** 1 IBM Research–Almaden, 650 Harry Rd., San Jose, CA 95120 USA; 2 IBM Research–Zurich, Säumerstrasse 4, Rüschlikon, 8803 Switzerland; 3 Department of Biosystems Science and Engineering, ETH Zurich, Mattenstrasse 26, 4058 Basel, Switzerland; 4 Present Address: Arctoris, 120E Olympic Avenue, Abingdon, OX14 4SA Oxfordshire, UK

**Keywords:** Polymer chemistry, Polymer synthesis

## Abstract

Advances in machine learning (ML) and automated experimentation are poised to vastly accelerate research in polymer science. Data representation is a critical aspect for enabling ML integration in research workflows, yet many data models impose significant rigidity making it difficult to accommodate a broad array of experiment and data types found in polymer science. This inflexibility presents a significant barrier for researchers to leverage their historical data in ML development. Here we show that a domain specific language, termed Chemical Markdown Language (CMDL), provides flexible, extensible, and consistent representation of disparate experiment types and polymer structures. CMDL enables seamless use of historical experimental data to fine-tune regression transformer (RT) models for generative molecular design tasks. We demonstrate the utility of this approach through the generation and the experimental validation of catalysts and polymers in the context of ring-opening polymerization—although we provide examples of how CMDL can be more broadly applied to other polymer classes. Critically, we show how the CMDL tuned model preserves key functional groups within the polymer structure, allowing for experimental validation. These results reveal the versatility of CMDL and how it facilitates translation of historical data into meaningful predictive and generative models to produce experimentally actionable output.

## Abstract (web summary)

Developing accessible polymeric materials is a challenging task for generative AI models. Here, the authors show that a domain-specific language enables the use of historical data to tune generative models to produce experimentally achievable material designs.

## Introduction

Artificial intelligence (AI) systems and machine learning (ML) models hold immense potential to accelerate development of polymeric materials by providing a significant reduction in time and labor costs to identify further optimized material platforms^1–5^. The combination of ML systems with automated experimentation platforms offers the possibility of realizing even greater reductions in research timelines^5–7^. The immensity of the potential benefits of ML systems for polymer science has resulted in intense development of models for a variety of use cases. These range from general inverse design of materials with given properties^8–13^ to specific applications including gas separation^14^, thermal conductivity^15^, mechanical toughness^16^, MRI contrast agents^17^, cloud point engineering^18^, and polymer electrolytes^19^. In several instances, the developed ML model was able to offer actionable material designs or predictions, leading to experimental validation of the model itself^12,17,18^. Although ML systems for polymer science are still in their early stages, it is clear they will play increasingly critical roles within research activities.

The routine application of ML models within polymer research workflows faces numerous obstacles. The absence of a mature open-source polymer data ecosystem has contributed to the limited data availability, accuracy, diversity, volume, and relevance for development of robust ML models^3,4,20^. Recent advances in open-source data repositories for polymeric materials such as the Polymer Genome or CRIPT will greatly assist in alleviating this issue as their respective data volume increases^21–23^. Historical data and data from automated experimentation systems can potentially provide both sufficient and immediately relevant training data for ML development, yet the data format and representation can be a significant barrier to utilization. Consequently, many reported ML models employ bespoke data models or polymer representations, which may not be readily adaptable to different application domains^17,18,24^. Improved data models for polymer chemistry have been developed to specifically address this issue^23,25,26^. However, software support for these models is still in the nascent stage and may require additional programming knowledge to utilize effectively. Our own work on compiling historical experimental datasets for polymer ML applications^12,27^ frequently required adding fields or elements that lay outside of the scope of existing data modeling approaches^23,25,26^ or traditional electronic lab notebooks^28,29^ in order to ensure accurate representation of the data. Thus, while open-source repositories, data models, and polymer representations have significantly advanced the development of ML models for polymer chemistry, there exists a need for software tools which provide flexibility in experimental data representations and their translation into ML training sets. Such tools would remove significant barriers for research groups to begin leveraging their own historical datasets in ML applications as well as provide an interface to the broader ecosystem of open-source tools, databases, and models being developed for polymer informatics.

**Fig. 1:**
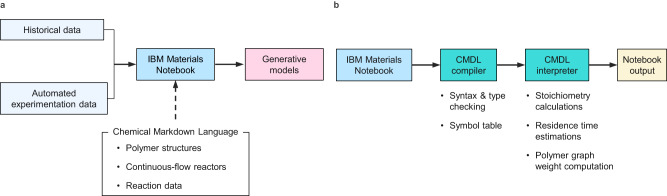
Enabling development of improved ML for guiding discoveries in polymer science. **a** The workflow for enabling consumption of historical and automated experimentation data in generative models using the IBM Materials Notebook and CMDL. **b** Workflow of how data is processed within the IBM Materials Notebook. Colors in both panels are for visual differentiation of each stage in the workflow.

To create a highly adaptable software toolkit for data representation and demonstrate its utility in ML workflows for catalyst and materials design (Fig. 1a), we first identified three critical features: (1) extensibility—such that new data or experiment types can be readily accommodated, (2) support for definition of polymer representations, and (3) support for representation of continuous-flow experiments. Initial efforts to implement such features within a web application, while successful, proved cumbersome to use and maintain. Taking a step back, we noted that the key requirement of extensibility is already a common feature in modern programming languages insofar as it enables users can define their own data structures and types to suit the needs of their application. Additionally, programming languages are parsed into abstract syntax trees (ASTs)^30^, a convenient intermediate data structure for further elaboration into formats required by ML pipelines. Because of these features, we surmised that a domain specific language (DSL)^31–33^ could be used as foundation of a software toolkit to enable data documentation upon which the other requirements—supporting polymer and continuous-flow reactor representations could be implemented.

## Results

### Domain-specific languages for data representation

Prior work on DSLs surrounding organic synthesis^33–38^, inorganic synthesis^39^, and biochemistry^40^ has focused on extracting action sequences from experimental protocols which in turn may be executed on compatible automated experimentation platforms^33,34,38^. In contrast, we sought to take advantage of benefits of DSLs to enable researchers to represent a broad variety of experiment data for use within AI-development pipelines, independent of the need for explicit execution on laboratory hardware. Herein, we developed the Chemical Markdown Language (CMDL) to provide a simple and declarative syntax for experimental documentation (see Supplementary Information for examples and overview of CMDL syntax). CMDL is an executable DSL and we deployed CMDL initially within a custom notebook extension for Visual Studio Code (VS Code)^41^—which provides an application programming interface (API) to define interactive computing applications analogous to JupyterLab^42^, Apache Zepplin^43^, and others^44,45^. The custom notebook extension—called IBM Materials Notebook^46^—provides a convenient execution environment for CMDL as well as allows users to leverage features of modern integrated development environments (IDEs)—such as code completion, templates, and snippets—to streamline documentation of experimental data using CMDL. Additionally, it enables researchers to use CMDL with minimal setup as the extension is easily installed from within VS Code.

Within the notebook extension, raw text written in CMDL is parsed and validated by the CMDL compiler into an AST (Fig. 1b). The valid CMDL ASTs are then passed to the CMDL interpreter, which traverses the ASTs and executes basic computations—such as reaction stoichiometry or residence time estimations—as well as performing tabulation and formatting of the data (Fig. 1b). The terms compiler and interpreter here are used loosely with regards to CMDL and simply refer to sections of the code performing static type checking and model execution, respectively. Once the CMDL has been compiled and interpreted, the final record may be exported in JavaScript object notation (JSON) for further aggregation into training data for ML models (Fig. 1b). An added advantage of CMDL is that it is relatively unopinionated on how experimental data should be organized, leaving these decisions to users. This is in contrast to more rigidly defined schema, such as PolyDat or CRIPT, which are geared more towards building large multi-user applications surrounding centralized databases^23,25^. Future versions of CMDL will maintain such flexibility while providing additional interoperability with data models such as PolyDat, CRIPT, and others when data are exported from CMDL notebooks.

### Polymer representation in CMDL

Definition and representation of polymer structures within experimental records is one of the key features of CMDL. The principal difficulty for polymer representation is that the stochastic nature of polymers precludes explicit definitions using line notations, such as Simplified Molecular Input Line Entry System (SMILES)^47^. In spite of this, there has been numerous efforts to define representations for polymers. Many reported ML models for polymers use SMILES strings denoting variable attachment points with an asterisk for repeat units^13,48^. BigSMILES provided a syntactical extension to the SMILES to allow broader representation of stochastic structures and end groups^49^. PolyGrammar was developed to facilitate both representation and generation of polymer structures through a context-sensitive grammar that combines a hypergraph representation and production rules to create polymer structures, however is currently implemented for only polyurethane structures^50^. Other approaches have focused on representing the polymeric structures as graphs, with nodes on the graph defined by SMILES and edges defining the stochastic connections^8,51,52^. For all polymer structural representation systems, its relationship with experimentally measured property values in the overall data structure is highly important for fully defining the stochastic properties of the polymer itself and establishing structure–property relationships^23,25^. With this in mind, were drawn to a graph representation approach for polymer structures as it would enable experimentally measured values—such as degree of polymerization (DP_n_)—to easily be embedded within the representation itself, allowing for differentiation of identical polymer structures on a basis of their stochastic properties. Moreover, using CMDL to define and reference polymer graph representations would allow users a simple and straightforward means to connect polymer structures (or components therein) with experimental conditions and property measurements—imparting potentially greater meaning and predictive capabilities to ML models.

**Fig. 2:**
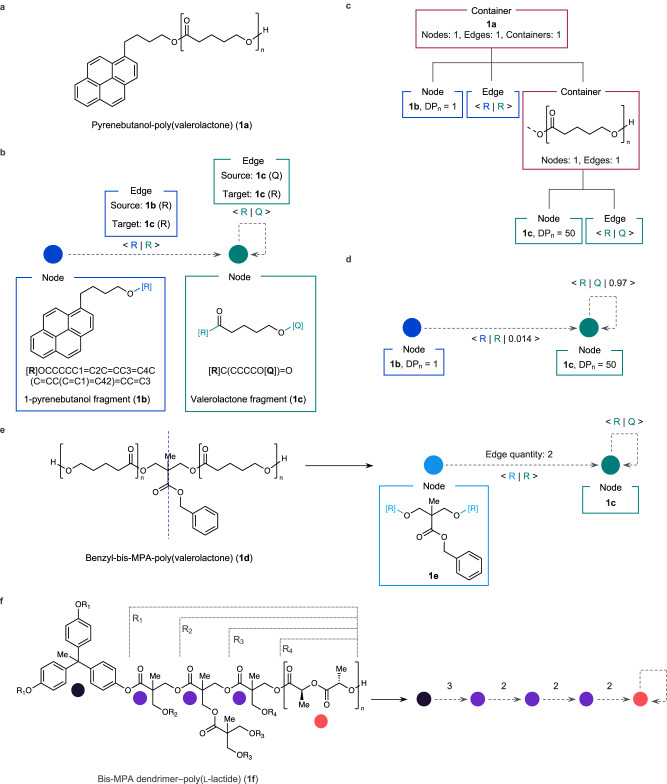
Graph representation of polymers. **a** Molecular structure of **1a**. **b** Schematic of a polymer graph representation of **1a**. Colored circles represent the polymer nodes within the graph with the corresponding boxes (labeled node) contain the SMILES fragment embedded within each node (**1b** or **1c**) along with the molecular structure with the attachment point ([R] or [Q]) highlighted in color corresponding to the node’s circle. The SMILES fragment within each node box highlights the attachment points in bold. Edges are represented by the grey dashed arrows with a label in angle brackets containing the source node and target node attachment points ([R] or [Q]) with the format of: <source attachment point | target attachment point >. The boxes labeled edge are color coded to match the source node from which the edge originates and contain the source and target nodes with their respective edge attachment points in parentheses. **c** A composite tree representation of a polymer graph for computing the edge weights of **1a**. Boxes are color coded to match their respective nodes or edge sources from **a** except container boxes which are colored red. Edge boxes contain the edge source node and target node information in the angle bracket form described in **b**. **d** Example of polymer edge weights computed from composite tree in **c**. Weights are listed as a third element in the edge angle bracket notation: <source attachment point | target attachment point | weight >. **e** Example of polymer graph compression using symmetry elements. Blue dashed line on **1b** represents a line of symmetry in the polymer. **f** Example of polymer graph compression for a dendritic polymer **1f**. Colored circles in skeletal structure represent distinct nodes in the polymer graph. Edge quantities for the graph are the numerical values listed above each edge (dashed grey arrow). Angle bracket notation for edges omitted for clarity.

To implement a polymer graph representation, a polymer structure may be deconstructed into the requisite nodes and edges which comprise a graph data structure. In this case, nodes represent discrete structural elements of polymer, such as an end group, repeat unit, or branch point. Edges correspond to a covalent bond or bonds between nodes. For example, poly(valerolactone) **1a** is comprised of two nodes, one for the 1-pyrenebutanol end group **1b** and one for the valerolactone repeat unit **1c** (Fig. 2a, b). The structure of each node element is encoded by a SMILES string containing non-atomic characters (R, Q, X, or Z) to distinguish different attachment points (Fig. 2b). Node elements containing multiple attachment points with identical chemical environments—such as structures with symmetric elements—are given same non-atomic character (Supplementary Fig. 19). Edges within the graph specify the source and target attachment point on the same node or between two different nodes (Fig. 2b). Assigning DP_n_ values to nodes allows for the computation of weights for the various edges within the graph representation, with the edge weight corresponding to the fraction of a particular edge (bond type) within the polymer graph. In Fig. 2c, d, the DP_n_ value of 50 is assigned to **1c** while a value of 1 is given to **1b**. These values allow computation of the weights for the two edges within the graph when the representation is processed by CMDL interpreter (see Supplementary Figs. 11–13 for examples).

The computation of the weights for polymer graphs is facilitated by their conversion to an intermediate composite tree representation within the CMDL compiler (Fig. 2c). The composite tree is a tree data structure whose leaf elements may comprised of nodes, edges, or containers. The node and edge elements within the composite tree are identical to those in the final polymer graph representation, whereas containers are elements that may have nodes, edges, or other containers as child elements (Fig. 2c). The use of a composite tree provides a more structured and chemically relevant means of programmatically traversing the polymer representation for computation of edge weights or other properties (Fig. 2d). This is especially important for more complex polymer architectures, such as dendrimers or grafted polymers, as it becomes difficult to consistently differentiate between main and side chains during traversal of the polymer graph, thereby complicating the accurate computation of edge weights. Instead, the hierarchy of the composite tree allows for clear differentiation between main and side chains within a polymer structure as well as nested repeating structure commonly found in many step-growth materials (Supplementary Figs. 21–22). Once the experimental values are assigned to different nodes, the intermediate composite tree representation can be used to recursively compute different edge weights.

While the CMDL syntax facilitates definition polymer graphs and assignment of experimental values to individual nodes, this approach can become tedious and repetitive when the polymer architecture becomes complex. To simplify this, we introduced a second edge weight indicating the quantity of identical edges within a polymer graph based on molecular symmetry. In Fig. 2e, the **1d** was initiated from a diol, providing two identical, yet distinct repeating nodes in the polymer graph. Rather than assigning DP_n_ values for each node individually, we can add an edge quantity weight to the edge between the diol initiator and the valerolactone repeat unit. This quantity weight is accounted for when the CMDL interpreter computes the weights for each edge within the graph representation. For more complex grafted or dendritic architectures (**1f**), this approach significantly reduces the number of nodes needing to be defined and assigned values (Fig. 2f).

### Representation of continuous-flow reactors in CMDL

**Fig. 3:**
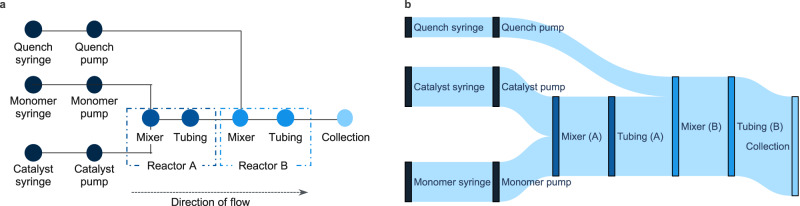
Graph representation of continuous-flow reactors. **a** Schematic representation of a continuous-flow reactor graph. Dotted boxes (Reactor A and Reactor B) denote elements belonging to a specific reactor wherein a chemical reaction takes place in the graph representation. Colors are used to differentiate nodes belonging to different reactors or the final terminal node in the reactor graph (Collection). See Supplementary Fig. 14 for an example of a reactor graph in CMDL syntax. **b** Sankey diagram reactor graph from **a**, where flow rate values have been assigned to the input nodes (Quench syringe, catalyst syringe, and monomer syringe) and propagated through the rest of the graph. Nodes in the Sankey diagram are color-coded to match those from Fig. 3a. Source data for **b** are provided as a Source Data file.

Along with representation of polymeric materials, the representation of experiments done under continuous-flow conditions are typically not supported in most data modeling efforts or traditional electronic lab notebooks^28,29^. Continuous-flow experiments are distinct from batch experiments and require modeling of the reactor system itself for accurate documentation. To facilitate this, continuous-flow reactors are represented as directed graphs whose nodes represent physical hardware components, edges the connections between the components, and the edge direction capturing the direction of flow (Fig. 3a). An analogous approach to reactor representation was developed for automated batch systems such as the Chemputer, although it was principally directed towards reactor process control^33^. As with polymer graphs, reactor graphs are defined separately (Supplementary Fig. 14) and referenced by other elements in the CMDL syntax, where inputs, outputs, and flow rates are assigned components of the reactor graph (Supplementary Fig. 16). A single run of a continuous-flow reactor may involve significant variation of the reactor conditions, such as changes in flow rates of the reactor inputs, and by extension, the residence time and stoichiometry of the reaction. Thus, each flow reaction group in the CMDL syntax represents a reaction conducted on single set of input conditions for a particular reactor. This simplifies the execution of models in the CMDL interpreter to propagate reagent flow through the reactor graph for stoichiometry calculations and estimation of residence times (Supplementary Figs. 16–17). Figure 3b depicts a Sankey diagram of a reactor graph with flow rates and their propagation through the reactor graph. While CDML representation of automated systems was initially focused toward continuous-flow systems, it can readily be extended to other high-throughput experimentation platforms. In these cases, a more abstract representation of the system and its inputs in CMDL syntax would be needed depending on its complexity. Future versions of CMDL will provide a means for defining these representations as well as automated processes actions and unit operations^33–36,38^—allowing a complete description of an automated high-throughput experimental process.

### Development and evaluation of regression transformer models

Having established the critical capabilities of CMDL and the encompassing notebook application, we could now better represent and merge historical experimental datasets and datasets from automated continuous-flow reactors. Next, we sought to utilize these data to develop ML models for assistance in catalyst and materials design. Our initial focus was on catalyst design for ROP, as generation of small-molecule catalyst structures and their experimental evaluation is more straightforward than that of material design. Additionally, catalyst selection is profoundly important for ROP and has dramatic effects on the overall viability of the ROP reaction^12,53,54^. Poor selection can lead to a mismatch between catalyst activity and monomer reactivity, giving either no polymerization or polymerization with poor control over end group fidelity and molecular weight distributions. Given the large breadth of catalysts and viable monomers for ROP, catalyst selection can be difficult—particularly for multiblock or statistical copolymers as co-monomers can exhibit very large differences in polymerizability^55,56^. Strategies such as using continuous-flow reactors or performing in situ catalyst switching can overcome some issues surrounding catalyst performance^57^. However, there exists a significant need for the development of catalysts for controlled ROP as well uncover critical design principles for more efficient organocatalysts in ROP, a task which can be greatly assisted through ML model development.

**Fig. 4:**
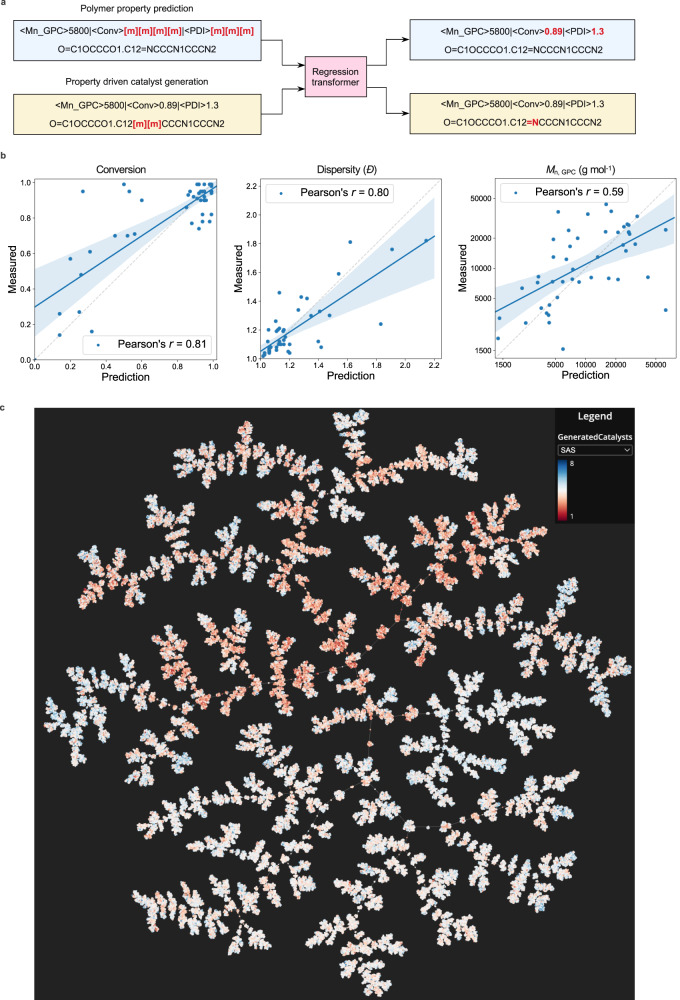
Generation of ROP using regression transformers. **a** Flowchart depicting training process for the regression transformer (RT). The RT can predict physical and experimental properties of monomer-catalyst pairs (blue stream) or conditionally generate catalysts given a monomer and desired properties (yellow stream). The SMILES input and output of the RT are shown here, however the RT internally uses SELFIES representations^71^. See the Methods section for details. **b** Prediction performance for conversion, dispersity, and *M*_n, GPC_ properties of monomer–catalyst pairs from the test data set (blue circles). Solid blue line is the linear regression fit, shaded blue area represents 95% confidence for the linear regression fit, and dashed grey line is hypothetical perfect fit. All Pearson correlations were statistically significant (*p* < 0.001; two-sided; normality assumption). *R*^2^ values are 0.66, 0.64 and 0.35 for conversion, dispersity and *M*_n, GPC_ respectively. The mean-absolute-errors are 0.12 (conversion), 0.10 (dispersity) and 0.26 (*M*_n, GPC_). Note that *M*_n, GPC_ has been modeled on a log_10_ scale. Conversion values are percentages plotted between 0 and 1, where 0 equals 0% conversion and 1 equals 100% conversion. **c** Tree manifold approximation and projection (TMAP) visualization^74^ of generated catalysts and their physical properties (colored here by synthesizability scores, SAS). Source data for **b** and **c** are provided as a Source Data file.

**Fig. 5:**
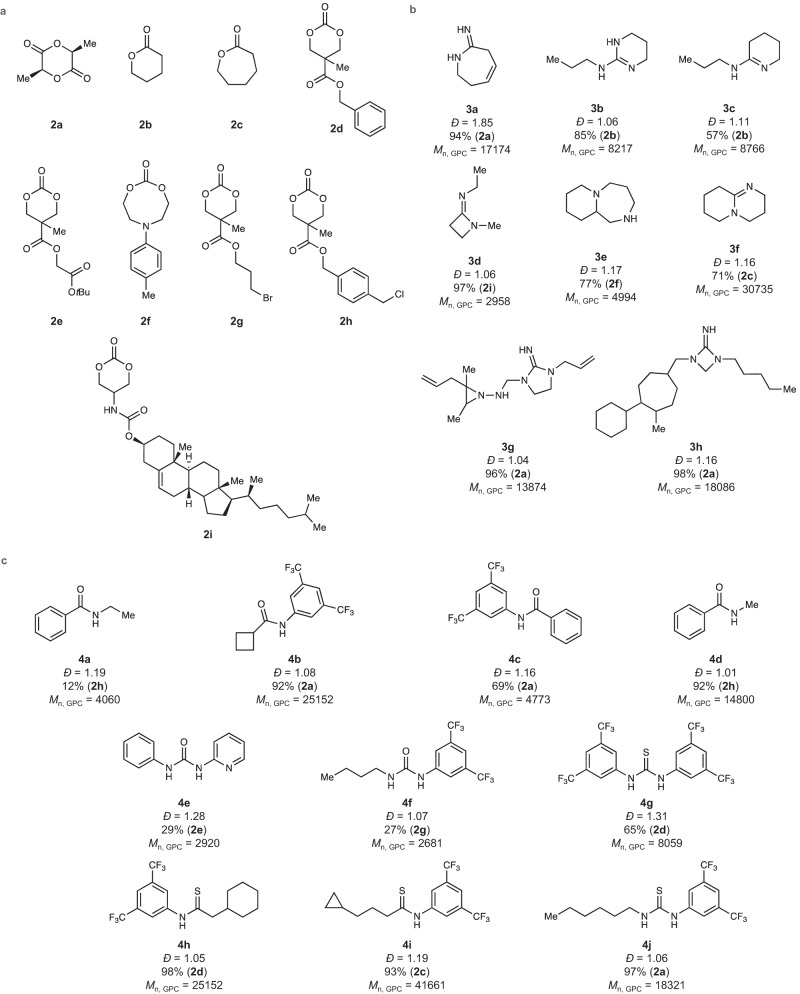
Selected generated ROP catalysts. **a** Monomers paired with ROP catalysts. **b** Selected generated nucleophilic/initiator activation ROP catalysts. Predicted values for dispersity, percent conversion of the paired monomer, and *M*_n, GPC_ are provided below each catalyst. **c** Selected generated electrophilic activation ROP catalysts. Predicted values for dispersity, percent conversion of the paired monomer, and *M*_n, GPC_ are provided below each catalyst. *M*_n, GPC_ values in **b** and **c** are in units of g mol^−1^.

To this end, we fine-tuned a property-driven a Regression Transformer (RT)^58^ generative model for local chemical space exploration (pretrained on ChEMBL data) with a ROP reaction dataset constructed from historical data using CMDL. The fine-tuned RT was then primed with monomers from the dataset and desired physical properties of interest and generated ≈2.5 M monomer–catalysts pairs (Fig. 4c). The RT was trained in a multitask fashion to regress conversion, dispersity and *M*_n, GPC_ from the SMILES strings of a monomer–catalyst pair (blue boxes, Fig. 4a) as well as to conditionally generate catalysts given a monomer and desired property values (yellow boxes, Fig. 4a). The RT learned to predict conversion and dispersity with high accuracy (Pearson correlation > 0.8). For monomer–catalyst pairs affording high conversion, the predictions were particularly accurate. The *M*_n, GPC_ prediction, performed on a log scale, was less successful (Pearson correlation 0.59). The lower predictive accuracy of absolute *M*_n, GPC_ values may be a result of biases within the historical dataset regarding monomer types and their influence on the resulting polymer’s hydrodynamic volume. This could potentially confound the RT learning to predict *M*_n, GPC_ values more so than conversion and dispersity, which are more independent of polymer identity. Given, these potential confounding factors, these property predictors were primarily used to filter and rank the generated catalysts to facilitate selection of promising candidates for experimental validation based on subject matter expert (SME) feedback (Fig. 5).

**Fig. 6:**
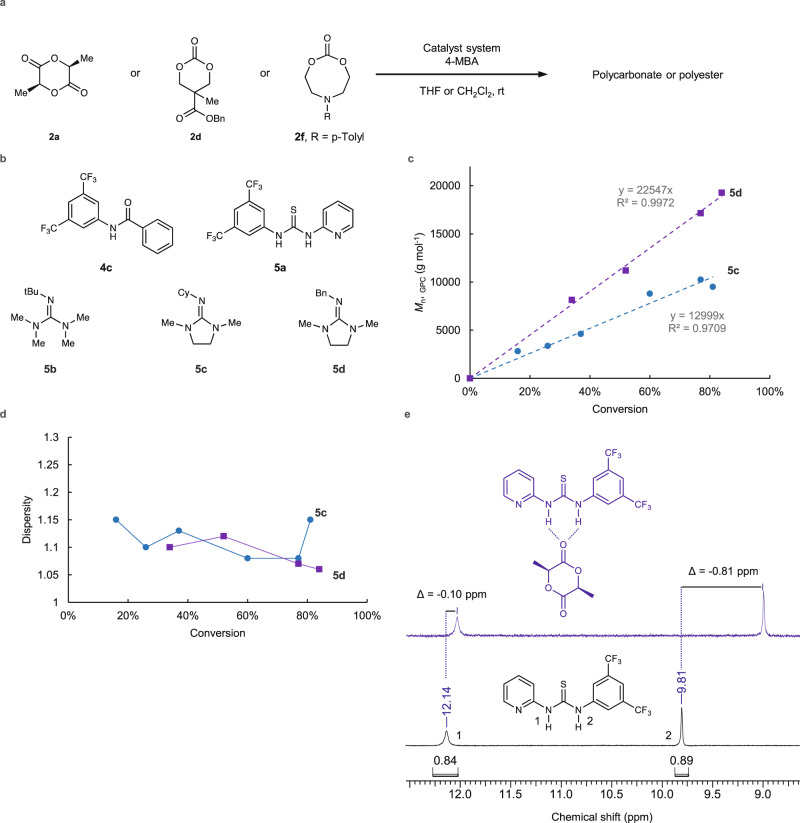
Experimental evaluation of generated ROP catalysts. **a** Scheme for polymerization reaction for experimental. **b** Selected and SME modified catalysts from Fig. 5 for experimental evaluation. **c** Plot of *M*_n, GPC_ versus monomer conversion for **5c** and **5d** in the polymerization of **2a**. **d** Plot of dispersity versus monomer conversion for **5c** and **5d** in the polymerization of **2a**. **e** Overlay of ^1^H NMR spectra in benzene-d6 showing the association of **5a** to **2a** and the observed in the change in chemical shift of the N–H protons of **5a**. The blue ^1^H NMR spectrum shows a mixture of **5a** and **2a**. The black ^1^H NMR spectrum shows **5a** alone with its N–H protons labeled with the numbers 1 or 2. See Supplementary Fig. 23 for ^1^H NMR association of **4c** with **2a**. 4-MBA = 4-methylbenzyl alcohol, DBU = 1,8-diazabicyclo[5.4.0]undec-7-ene. Source data for **c** and **d** are provided as a Source Data file.

Of the SME selected examples in Fig. 5b and c, only **3b** and **4g** have been previously reported as catalysts for ROP^54,59^, whereas the amidine, amide, guanidine, urea, and thiourea motifs present in the other generated catalysts are common components of known ROP catalysts^53,60–63^. Despite the common structural features, it is well known that small structural modifications to ROP organocatalysts can drastically affect reaction kinetics, selectivity, and control over the polymerization^59,60,64^. Thus, the generated catalysts are highly important in identifying potentially useful catalyst platforms which may offer significant improvements over existing systems. Experimental evaluation of the catalyst structures as generated may not be practical in all cases, particularly where the structure is somewhat complex. To test the viability of some of the generated catalysts, we chose five catalysts, four of which were modified by SMEs from their original form to expedite evaluation of their catalyst properties. Here, **5a** (Fig. 6) was selected as a modified version of **4e** to improve possible solubility issues of the catalyst during ROP. Thiourea catalyst **5a** has only been reported for use as ROP catalyst with highly reactive *o*-carboxyanhydrides^65^, yet is untested in polymerizations with lactones or cyclic carbonates. Catalysts **5c** and **5d** (Fig. 6) were selected both on terms of straightforward synthetic accessibility as well as serving as surrogates for the generated catalyst **3g**, preserving the endocyclic guanidine moiety. Commercially available catalyst **5b** serves as a contrasting, acyclic guanidine catalyst to **5c** and **5d**. The cyclic versus acyclic nature of guanidine catalysts for ROP is known to influence catalyst reactivity and thus is important to include both here for experimental validation^64,66^.

The selected catalysts selected for experimental validation can largely be split between hydrogen bond donors (**4c** and **5a**) as electrophilic activators of the monomer or Brønsted bases as nucleophilic activators of the initiator (**5b**–**5d**, Fig. 6), none of which have previously been utilized in ROP. While some hydrogen bond donors may act as single-component catalysts for ROP, this is typically only for instances where such systems contain a pendant tertiary amine^67^. With the case of **5a**, the attached pyridine group did not provide sufficient activation of the alcohol initiator to facilitate polymerization by itself, despite NMR experiments showing a strong affinity association of **5a** with **2a** (Fig. 6c). Instead, use of a DBU co-catalyst was needed to enable ROP of both **2a** and **2d** (entries 1 and 8, Table 1). In the case of **2d**, comparison of the use of DBU alone to DBU with **5a** demonstrated that the addition of **5a** provided greater control over the dispersity without slowing down the polymerization reaction (entries 6 and 8, Table 1). The guanidine derivatives **5b**–**5d** also performed well as single component ROP catalysts, providing high monomer conversion and narrow dispersity (entries 3–5, 9, and 10, Table 1). Both **5c** and **5d** displayed a linear relationship between *M*_n, GPC_ versus monomer conversion (Fig. 6c), characteristic of living ROP^53^.

**Table 1:** ROP data from experimental evaluation of generated catalysts

Entry	Monomer	[M]_0_:[I]_0_^a^	Catalyst	Time	Conversion (%)	*M*_n, GPC_ (kg mol^−1^)	*Đ*
1	2a	60	5a + DBU	18 h	100	24.9	1.51
2	2a	60	4c + DBU	18 h	>95	9.7	1.48
3	2a	60	5b	4 min	100	14	1.15
4	2a	60	5c	32 min	60	9.8	1.06
5	2a	60	5d	190 min	84	19.3	1.06
6	2d	50	DBU	30 min	99	13.7	1.24
7	2d	50	5a	30 min	0	—	—
8	2d	50	DBU + 5a	30 min	99	18.4	1.06
9	2d	60	5c	45 min	100	4.7	1.34
10	2 f	60	5b	2 min	100	16	1.21

^a^Initial monomer (M) to initiator (I) ratio.

**Fig. 7:**
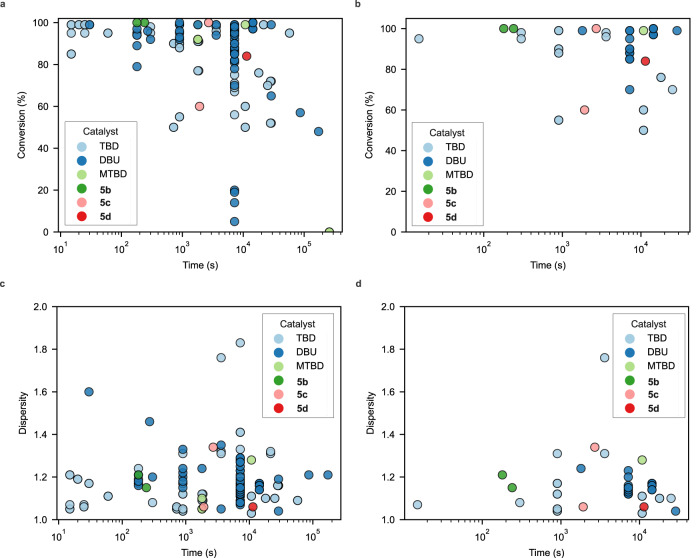
Comparison of historical ROP reaction data for with generated catalysts. **a** Historical data for monomer conversion versus reaction time for single component guanidine and amidine catalyzed ROP reactions for all targeted DP_n_. **b** Historical data for monomer conversion versus reaction time with a targeted DP_n_ ≈ 50, similar to experiments from Table 1. **c** Historical data for dispersity versus reaction time for single component guanidine and amidine catalyzed ROP reactions for all targeted DP_n_. **d** Historical data for dispersity versus reaction time for single component guanidine and amidine catalyzed ROP reactions with a targeted DP_n_ of ≈ 50, similar to experiments from Table 1. In all plots, the reaction time is plotted on a log_10_ scale. Source data for all plots are provided as a Source Data file.

By having a historical dataset for ROP reactions with a variety of monomers, we can make some broader comparisons of the reactivity of the generated catalysts and similar catalysts from the historical dataset assembled in CMDL and used to fine-tune the catalyst RT. Here, we compared the historical results of triazabicyclodecene (TBD), DBU, and 7-Methyl-1,5,7-triazabicyclo(4.4.0)dec-5-ene (MTBD) with the guanidine catalyst systems **5b**–**5d** in terms of monomer conversion, reaction time, and the resulting dispersity of the polymeric material. Based on these metrics, we can see that all the generated guanidine bases are clearly comparable to traditional catalysts both in terms of reaction time and control over the molecular weight distribution (Fig. 7). Of particular interest is the clear difference in reactivity between the cyclic guanidine **5c** and the acyclic analogue **5b**, where **5b** exhibits faster kinetics and comparable control over dispersity. This behavior is consistent with previously observed trends between cyclic and acyclic guanidines in ROP and other reactions^66,68^.

**Fig. 8:**
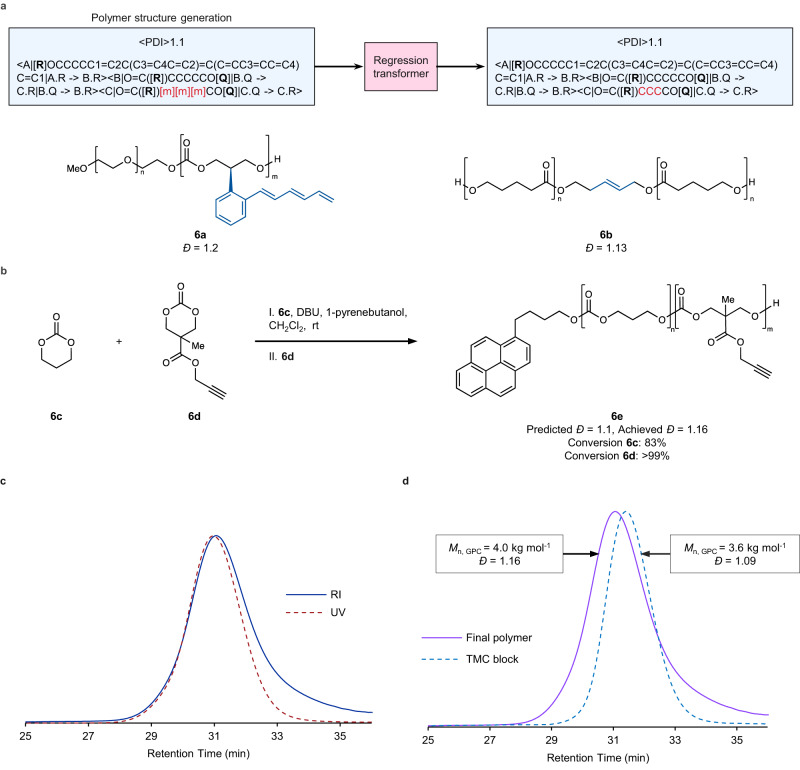
Generation and evaluation of polymers with regression transformers using CMDL polymer graph representations. **a** General flowchart (blue boxes) depicting training process for the polymer graph RT. In the blue boxes, the dispersity property (encoded as <PDI > ) and the polymer graph string is listed below. As in Fig. 2, the non-atomic placeholder characters in the SMILES string are set in bold and enclosed in brackets. See Supplementary Fig. 26 for an explanation of the polymer graph string syntax. **6a** and **6b** are examples of the generated polymer structures from the RT. **b** Reaction schema for the experimental evaluation of generated polymer structure **6e**. **c** Overlay of GPC traces recorded RI and UV detection. **d** Overlay of GPC traces from the first block—TMC block (**6c**)—and the final polymer (**6e**). Data for GPC traces in **c** and **d** have been normalized. Source data for **c** and **d** are provided as a Source Data file.

Having successfully demonstrated the RT model for development of polymerization catalysts, we next sought to leverage a similar model for the design of polymeric materials. The use of inverse design approaches or generative models for identification of polymeric materials with improved properties are becoming increasingly important for guiding experimental research for polymeric materials^1,11,15,16,69^. Much of the focus of generative modeling for polymer structures has been on homopolymers^11,15,69^ or simple copolymers derived from polycondensation or polyaddition reactions^16,50^. Additionally, relatively few of these studies have carried out subsequent experimental synthesis and validation of the generated structures^16^. Thus, while existing reports are successful in generating new repeat units for polymer structures, there is no guarantee that these generated polymers are experimentally accessible. This is especially true if the chemical environment of the attachment points in a repeat unit SMILES string have been modified by the model—potentially precluding the newly generated repeat unit from known polymerization reactions. In contrast to small-molecule synthesis, polymerization reactions tend to have more stringent requirements in order to realize successful enchainment of monomeric repeat units. In the case of ROP, it is well understood that small changes in monomer structure can dramatically alter its thermodynamic parameters for polymerization, potentially rendering it impossible to enchain^55,56^. To address this, we felt that the CMDL polymer graph representation in combination with a more restricted RT model—where modifications are limited to certain segments of a particular structural entity in a node or edges between nodes (Fig. 8a)—would provide a means for both preserving the repeat unit attachment points as well as generate more complex architectures than reported by previous approaches.

Although CMDL represents polymers as graphs natively, the CMDL interpreter can readily serialize them, enabling their consumption within language models (Fig. 8a, Supplementary Fig. 26). Using these polymer graph strings and their dispersity values from the experimental ROP dataset, we fine-tuned a modified RT model to produce over 2500 polymer structures focused primarily on block and statistical copolymers (Fig. 8a). The generated polymer graph strings were parsed into CMDL syntax and embedded within a notebook document for inspection and selection of promising candidates by SMEs for experimental evaluation (Fig. 8a, Supplementary Fig. 18). Upon inspection, many of the generated polymer structures contained invalid SMILES strings. These SMILES strings were either incomplete—such as missing a parenthesis—or produced chemically invalid structures. Other generated structures simply reproduced the training data with no modification, which is not unexpected given the more restricted masking approach used in this RT model. Finally, several generated polymer structures contained invalid repeating chemistry. Aside from these examples, there were a substantial number of examples where the model performed precisely as intended—modifying only certain portions of the discrete structural entities in the nodes of the polymer graph.

The successful examples typically fell into three categories: modification of the repeat units, modification of the initiator, or recombination of existing repeat units into (co)polymer architectures. Selected examples can be found in Fig. 8a and Supplementary Figs. 28 and 29, along with their predicted dispersity. For both the strict modification of the monomer (**6a**, Fig. 8a and Supplementary Fig. 24) and modification of the initiator (**6b**, Fig. 8a, Supplementary Fig. 25a), the model performed admirably by making targeted modifications to the overall polymer structure while preserving the connectivity between repeat units. For the examples of generating (co)polymer architectures from existing repeat units, it is likely many of these were produced by a single atom modification to give a different repeat unit that also existed in the training data. For example, the trimethylene carbonate repeat unit in generated polymer **10c** (Supplementary Fig. 25b) was likely produced via conversion of the alpha methylene carbon of valerolactone to an oxygen atom, converting the ester repeat unit into a carbonate. Additionally, several instances were observed where the model produced a somewhat ambiguous assignment of connectivity between the nodes of the generated polymer graph (**6e**, Fig. 8b and **10a**, Supplementary Fig. 25b). In these cases, we interpreted the polymer architecture as the corresponding AB block copolymer architectures, given that the statistical copolymer versions were part of the training data and were also correctly reproduced by the model output.

Although polymer structures containing new repeat units can potentially afford and desirable properties, they can also require significant time and effort realize experimentally. As noted above, new monomer structures may have profoundly different abilities to undergo ROP as a result of changes in their thermodynamic parameters. This change in reactivity also influences the choice of catalyst, necessitating careful selection in order to avoid deleterious side-reactions during polymerization. With these factors in mind, we selected polycarbonate block copolymer **6e** (Fig. 8b) for experimental evaluation as both monomers are present in the training data, but not in this particular block copolymer architecture. Here, using DBU as catalyst, we were able to smoothly prepare **6e** in a single pot transformation using 1-pyrenebutanol as an initiator (Fig. 8b). Overlay of GPC traces using refractive index (RI) and ultraviolet (UV) detection showed good agreement (Fig. 8c), indicating high end group fidelity. Additionally, GPC traces of the first block and the final polymer showed an increase in molecular weight and minimal broadening of the molecular weight distribution (Fig. 8d). Notably, the predicted and realized dispersity (1.10 vs. 1.16) are very close, and better agreement could likely be obtained through optimization of the reaction time for the more reactive monomer (**6d**, Fig. 8b).

**Fig. 9:**
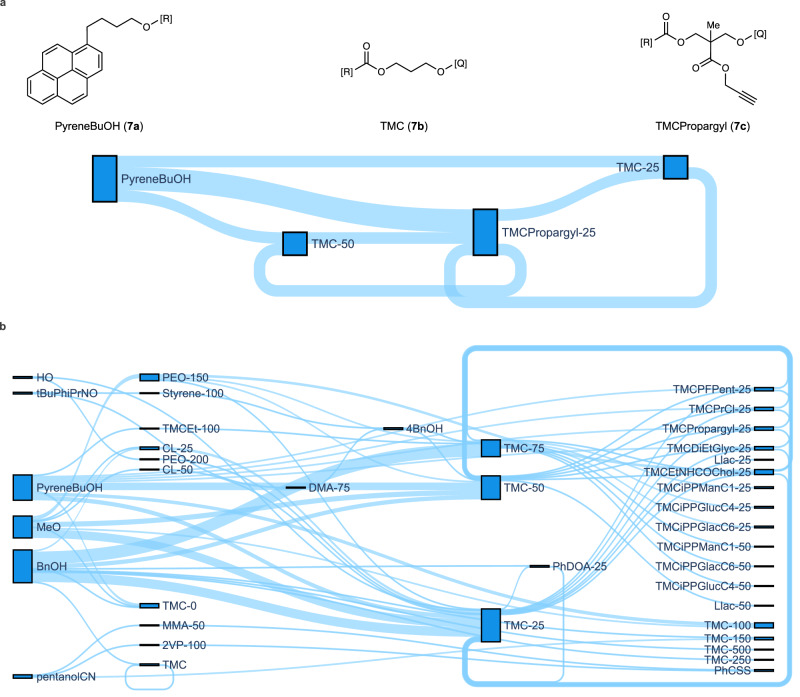
Sankey visualization of grouped polymer graphs from historical experimental data. **a** Visualization for all polymer graphs in training data containing **6d**. **b** Visualization of all polymer graphs in training data containing **6c**, see Supplementary Fig. 27 for corresponding structures for the Sankey nodes. Black outlined, blue boxes are nodes within the Sankey visualization and light blue paths between nodes are the links. The width of the link corresponds to the number of materials in the historical data containing the corresponding edge in the polymer graph representation. In both **a** and **b**, numerical suffixes (e.g., −25 or −100) on the Sankey node label indicate the bin value for the experimentally assigned DP_n_ of that element within the polymer graph. A value of 0 indicates a failed polymerization reaction (e.g., TMC-0). No suffix indicates the element was not a repeat unit (no self-referencing edge, such as MeO or BnOH) or where no DP_n_ information was available (TMC). Source data for both plots are provided as a Source Data file.

While the RT generative model was successful in producing valid polymer structures, it can be difficult to place the predicted structures in the context of the historical training data. One of the major advantages of the CMDL graph representation is that enables the embedding of experimentally measured DP_n_ values within nodes of the representation itself (Fig. 2b, c). Using this feature within the polymer graph representation system, we can group polymer graphs from the historical data based on common structural entities and the edges between them. Entities with embedded DP_n_ values can be further split into buckets based on ranges for DP_n_, including a bucket for DP_n_ equal to 0, indicating a failed polymerization. Once the polymer graph data has been grouped, it may be visualized as a Sankey diagram (Fig. 9), with the width of each node or edge indicating the number of materials in the dataset with that particular entity or connection between entities, respectively. In Fig. 9, two Sankey diagrams are shown for all polymer graphs containing either **6d** (Fig. 9a) or **6c** (Fig. 9b). These visualizations help provide a broad overview of the types of materials prepared, the range of assigned DP_n_ values, and their interconnectivity. For **6c**, the monomer has been frequently used within the historical dataset within a variety of material types (Fig. 9b). In contrast, **6d** has only been used in materials with **6c** and 1-pyrenebutanol (Fig. 9a). Notably, some of the generated polymers containing **6c** or **6d** as repeat units (**8** **f** and **8** **g**, Supplementary Fig. 24; **10c**, Supplementary Fig. 25b), the connectivity between their corresponding structural elements is not present in the Sankey diagrams (Fig. 9), indicating again the value of the RT in generating new, viable polymer structures.

## Discussion

The ability to routinely employ ML models for day-to-day research activities will have a dramatic impact on the research and development of polymeric materials. However, to effectively develop and use ML in experimental research, there must be a straightforward way to easily leverage relevant experimental data from a variety of sources. Herein we have demonstrated how CMDL and its implementation within the IBM Materials Notebook extension can serve as a platform to represent and merge disparate experimental data types. In particular, the built-in support for graph representation of polymeric structures and continuous-flow reactors allows for straightforward use of these representation systems and their connection to experimental data. In turn, datasets created through use of CMDL facilitated the development of highly effective RT models for the design of ROP catalysts and architecturally valid co-polymers. Successful experimental evaluation of the generated catalyst structures and preparation of a generated block co-polymer demonstrate the utility of these models in providing both actionable and useful output. Importantly, the use of CMDL polymer graph representations facilitated the generation of more experimentally viable polymer candidates through the preservation of critical functional groups. Expansion and refinement of this approach will likely afford significant advances in ML-designed polymeric materials with optimized properties which are also experimentally accessible. Overall, CMDL and its application within experimental research workflows provides a highly adaptable tool for enabling researchers to use historical experimental data for the development of more meaningful and impactful ML models.

## Methods

### IBM Materials Notebook

IBM Materials Notebook is an open-source extension written in TypeScript for Microsoft’s Visual Studio Code (VS Code)^41^. The extension provides support for a custom notebook allowing for composing and executing CMDL using VS Code’s notebook extension API (https://code.visualstudio.com/api) Visualization of chemical structures within the IBM Materials Notebook extension was accomplished using a TypeScript implementation of the SmilesDrawer package (https://github.com/reymond-group/smilesDrawer). Tutorials, examples, and additional documentation for IBM Materials Notebook and CMDL can be found at the repository documentation website (https://ibm.github.io/ibm-materials-notebook/).

### Data Visualization

Historical experimental data was written in CMDL, exported as JSON files using the IBM Materials Notebook built-in export command, and subsequently imported to a local instance of MongoDB (https://www.mongodb.com). This database was queried for data for Figs. 7 and 9 using Pymongo (https://pymongo.readthedocs.io/en/stable/), the Python database driver for MongoDB, and imported to a Jupyter Notebook (within a JupyterLab^42^ environment) where the data was transformed using Pandas (https://pandas.pydata.org/) and plotted using the Seaborn^70^ and Plotly (https://plotly.com/python/) libraries.

### Fine-tuning process for the catalyst model

Following the original implementation and hyperparameters of the RT, the drug-likeness (QED) model as described by Born & Manica^58^ was taken as starting point. This model had been pre-trained on 1.4 million molecules from ChEMBL (https://www.ebi.ac.uk/chembl/) with drug-likeness as single property with two alternating training objectives of predicting a sequence of characters corresponding to the continuous QED value (thus performing a regression task) and a generative objective where the RT had to reconstruct a full molecule given a corrupted (i.e., partially masked) molecule as well as its continuous property value. We then finetuned this RT on 549 monomer–catalyst pairs, each associated to three physical and experimental properties (monomer conversion, dispersity, and *M*_n, GPC_). During training, the RT alternated between predicting the properties for monomer–catalyst pair and generating catalysts given a monomer and desired properties. We used a learning rate of 0.0002, a batch size of 8 with 5 steps for gradient accumulation. The two tasks were alternated every 50 steps. In the generative task, 40% of the catalyst tokens were masked, the monomers were not masked. The maximal span length was 7 tokens. We used the self-consistency loss as proposed by Born & Manica^58^ since it produces superior performance for generative tasks (in exchange to slight performance loss on predictive tasks). The property prediction results reported in Fig. 6b were produced on a held-out test set of 61 samples. Catalysts and monomers were represented as SELFIES^71^ rather than SMILES strings (to ease the generative task) and data augmentation^72^ was used with a factor of 32 on the training dataset to boost model generalization.

### Fine-tuning process for the polymer model

For the block copolymer experiments, we leveraged the RT model pretrained on 2.8 million chemical reactions from USPTO, represented as SMILES sequences as described in the work by Born and Manica^58^. This model was then finetuned on the polymer graph representation, wherein each node consisted of a SMILES fragment, of 1566 polymers, each associated to two experimental properties (dispersity and *M*_n, GPC_). The polymers were represented by a sequence representation derived from the CMDL. To the best of our knowledge, it thus constitutes the first generative model for block copolymers. Like for the catalyst model, the RT training alternated every 50 steps two tasks, in this case predicting the properties and completing partially masked block copolymers given the property scores. In the generative task, 30% of the tokens of the block copolymers were masked and the maximal span length was again 7 tokens. No data augmentation was used. The learning rate was 0.002 and the batch size was again 8. All models were trained on a single Nvidia A100/V100 GPU for less than a day. The generated polymers were converted to CMDL and inspected using the IBM Materials Notebook prior to selecting a candidate for experimental validation, see Supplementary Fig. 18 for an example.

### General procedure for ROP experiments

In a nitrogen-filled glovebox, a vial containing a magnetic stir-bar was charged with 4-MBA (1 eq), catalyst (2.5 eq), and co-catalyst (2.5 eq, if needed) were dissolved in CH_2_Cl_2_ (0.9 mL). Under vigorous stirring, a solution of monomer (50 eq) in CH_2_Cl_2_ was added via syringe to the vial containing the initiator/catalyst solution. Aliquots were taken periodically and quenched with 0.1 mL of a benzoic acid (24.4 mg, 5 eq) solution in CH_2_Cl_2_ (0.5 mL). Solvents were evaporated under reduced pressure and crude samples analyzed by GPC and ^1^H NMR. Following completion of the reaction, excess benzoic acid was added, and the reaction mixture was removed from the glovebox. The polymer was purified by precipitation into isopropanol (45 mL), followed by centrifugation (1132×*g*) and decantation of the supernatant. This process was repeated 3 times. The isolated polymer was dried *in vacuo* before characterization via GPC and NMR.

### Polymerization of 2a (Table 1, entry 3)

Following the general procedure, 4-MBA (4.9 mg, 0.04 mmol, 1 eq), **5b** (5.2 mg, 0.03 mmol, 1 eq), **2a** (288 mg, 2 mmol, 50 eq), and CH_2_Cl_2_ (1 mL) were reacted for 4 min. The title compound was isolated after workup and purification as described in the general procedure. *M*_n_ (GPC): 14 kg mol^−1^ Dispersity: 1.15 ^1^H NMR (400 MHz, CDCl_3_): δ (ppm): 5.17–5.15 (m, 2H), 1.59–1.58 (m, 6H).

### Polymerization of 2d (Table 1, entry 8)

Following the general procedure, 4-MBA (4.9 mg, 0.04 mmol, 1 eq), DBU (15.2 mg, 0.1 mmol, 2.5 eq), **5a** (36.5 mg, 0.1 mmol, 2.5 eq), **2d** (500 mg, 2 mmol, 50 eq), and CH_2_Cl_2_ (1 mL) were reacted for 30 min. The title compound was isolated after workup and purification as described in the general procedure. *M*_n_ (GPC): 18.4 kg mol^−1^; Dispersity: 1.06; ^1^H NMR (400 MHz, CDCl_3_): δ (ppm): 7.29 (m, 102H), 7.16 (m, 2H), 5.12 (m, 40H), 5.10 (s, 2H), 4.27 (m, 74H), 2.33 (s, 3H), 1.22 (s, 58H).

### Polymerization of 2 f (Table 1, entry 10)

Following the general procedure, 4-MBA (4.9 mg, 0.04 mmol, 1 eq), **5b** (36.5 mg, 0.1 mmol, 2.5 eq), **2** **f** (265 mg, 1.2 mmol, 30 eq), and CH_2_Cl_2_ (1 mL) were reacted for 30 min. The title compound was isolated after workup and purification as described in the general procedure. *M*_n_ (GPC): 16 kg mol^−1^; Dispersity: 1.21; ^1^H NMR (400 MHz, CDCl_3_): δ (ppm): 7.03 (m, 65H), 6.55 (m, 65H), 5.09 (m, 2H), 4.22 (m, 124H), 3.57 (m, 127H), 2.34 (s, 3H), 2.22 (m, 92H).

### Synthesis of 6e (Fig. 8b)

In a nitrogen filled glove box, a 5 mL vial was charged with 1-pyrenebutanol (0.0269 g, 0.09 mmol), DBU (0.014 g, 0.09 mmol), and CH_2_Cl_2_ (0.25 g). **6c** (0.30 g, 2.90 mmol), was dissolved in CH_2_Cl_2_ (0.8 g) and added to the vial containing 1-pyrenebutanol and DBU. The reaction mixture was stirred at rt and aliquots were periodically removed as to assess **6c** conversion by ^1^H NMR. After reaching 86% conversion of **6c** (95 min), **6d** (0.32 g, 1.74 mmol) was dissolved in CH_2_Cl_2_ (0.8 g) and added to the reaction mixture. Aliquots were removed to monitor the reaction progress and after full conversion of **6c**, excess benzoic acid was added to quench the polymerization. The reaction mixture was removed from the glovebox and the copolymer was purified by precipitation in isopropanol (45 mL), centrifugation (1,132 × *g*), and decantation of the supernatant. The isolated solid was subsequently dried *in vacuo*. *M*_n_ (GPC): 4.0 kg mol^−1^; Dispersity: 1.16; ^1^H NMR (400 MHz, CDCl_3_): δ (ppm): 4.73 (s, 2H), 4.24 (m, 11H), 2.05 (m, 4H), 1.29 (s, 2H).

### Reporting summary

Further information on research design is available in the Nature Portfolio Reporting Summary linked to this article.

## Supplementary information

Supplementary Information

Reporting Summary

## Data Availability

The data used and generated in this study can be found at the IBM Materials Notebook GitHub repository at https://github.com/IBM/ibm-materials-notebook/tree/main/data^46^. Source data are provided with this paper.

## Source data

Source Data

## Code availability

Code for CMDL and the IBM Materials Notebook are available at the GitHub repository under https://github.com/IBM/ibm-materials-notebook^46^. The VS Code extension for IBM Materials Notebook is freely available for download and use from the VS Code extension marketplace at https://marketplace.visualstudio.com/items?itemName=IBMMaterials.ibm-materials-notebook. The regression transformer models can be found at https://huggingface.co/spaces/GT4SD/regression_transformer^73^.
