# Author Correction: Artificial intelligence driven design of catalysts and materials for ring opening polymerization using a domain-specific language

**DOI:** 10.1038/s41467-023-40277-y

**Published:** 2023-07-25

**Authors:** Nathaniel H. Park, Matteo Manica, Jannis Born, James L. Hedrick, Tim Erdmann, Dmitry Yu. Zubarev, Nil Adell-Mill, Pedro L. Arrechea

**Affiliations:** 1 IBM Research–Almaden, 650 Harry Rd., San Jose, CA 95120 USA; 2 IBM Research–Zurich, Säumerstrasse 4, Rüschlikon, 8803 Switzerland; 3 Department of Biosystems Science and Engineering, ETH Zurich, Mattenstrasse 26, 4058 Basel, Switzerland; 4 Present Address: Arctoris, 120E Olympic Avenue, Abingdon, OX14 4SA Oxfordshire, UK

**Keywords:** Polymer chemistry, Polymer synthesis

Correction to: *Nature Communications* 10.1038/s41467-023-39396-3, published online 21 June 2023

The original version of this Article contained an error in Table 1, where the seventh and eighth entries in the first column were both given as ‘8’. The seventh entry has been changed to the correct value ‘7’ in the corrected version.

In addition, three references to table entries were given incorrectly in the main text. In the fifth sentence of the fourth paragraph in the section ‘Development and evaluation of regression transformer models’ of the Results, the reference to Table 1 was incorrectly given as ‘entries 5 and 8, Table 1’ instead of the correct ‘entries 6 and 8, Table 1’. The seventh and eighth section titles of the Methods were incorrectly given as ‘Polymerization of 2d (Table 1 entry 7)’ and ‘Polymerization of 2f (Table 1, entry 9)’ instead of the correct ‘Polymerization of 2d (Table 1, entry 8)’ and ‘Polymerization of 2f (Table 1, entry 10)’. These errors have been corrected in both the PDF and HTML versions of the Article.

