# Supplementary material for: Artificial intelligence driven design of catalysts and materials for ring opening polymerization using a domain-specific language
Source: Nat Commun. 2023 Jun 21;14:3686. doi: 10.1038/s41467-023-39396-3 (PMC10284867; doi:10.1038/s41467-023-39396-3)
Supplement: Supplementary file 1 — Supplementary Information [file 41467_2023_39396_MOESM1_ESM.pdf]

Supplementary Information

Artificial intelligence driven design of catalysts and materials for ring opening polymerization using a domain-specific language

Nathaniel H. Park, Matteo Manica, Jannis Born, James L. Hedrick, Tim Erdmann, Dmitry Yu. Zubarev, Nil Adell-Mill, Pedro L. Arrechea

Correspondence to: npark@us.ibm.com

Supplementary Methods

General material information. 2,2-bis(hydroxylmethyl) propionic acid (98%, Sigma-Aldrich), *N,N*-diisopropylethyl amine (DIEA, >98%, Sigma-Aldrich), 3,5-bis(trifluoromethyl aniline) (97%, Sigma-Aldrich), *N,N'*-carbonyldimidazole (CDI, >98%, Oakwood Chemical), 3,5-Bis(trifluoromethyl)phenyl isocyanate (98%, Sigma-Aldrich), 2-aminopyridine (>99%, Sigma-Aldrich), *N,N,N',N'*-tetramethylethylenediamine (TMEDA, 99%, Oakwood Chemical), benzyl bromide (BnBr, 98%, Sigma-Aldrich), 2,2′-(4-methylphenylimino)diethanol (90%, Sigma-Aldrich), propargyl bromide (80 wt. % in PhMe, Sigma-Aldrich), *p*-toluenesulfonyl chloride (TsCl, 99%, Oakwood Chemical), 2-chloro-1,3-dimethylimidazolinium chloride (98%, Oakwood Chemical), cyclohexylamine (99%, Sigma-Aldrich), benzylamine (99%, Sigma-Aldrich), benzoyl chloride (99%, Sigma-Aldrich), potassium phosphate tribasic (98%, Sigma-Aldrich), 1-pyrenebutanol (99%, Sigma-Aldrich), L-lactide (**2a**, 99%, Purac). 4-methylbenzyl alcohol (4-MBA, 98%, Sigma-Aldrich) was sublimed prior to use and stored in an N_2-filled glovebox. 1,8-diazabicyclo[5.4.0]undec-7-ene (DBU, 98%, Sigma-Aldrich) was distilled prior to use and stored in an N_2-filled glovebox. Trimethylene carbonate (**6c**, 99%, Purac) was purified by dissolving in dichloromethane, filtration, and then recrystallized by dissolution in a minimal amount of dichloromethane, adding Et_2O until the cloud point, and then cooling the mixture to -20 °C in a freezer. Crystals of **6a** were collected via filtration and washing with additional cold Et_2O. All polymerization reactions were performed in a nitrogen-filled glovebox using anhydrous solvents. All commercially available materials were purchased from Sigma-Aldrich or Oakwood Chemical and used as received unless otherwise specified.

General analytical information. All ^1H NMR spectra were collected at room temperature using a Bruker Avance NMR Spectrometer operating at 400 MHz. All ^{13}C spectra were collected at room temperature using the same instrument operating at 100 MHz. ^1H and ^{13}C NMR spectra were referenced to the internal residual solvent signal (7.26 ppm and 77.16 ppm, respectively for $CDCl_3$). Molecular weight distributions of all polymers were determined by using a Waters system equipped with four 5 μm columns (300 mm × 7.7 mm) connected in series (with increasing pore sizes: 100, 1000, 10^5, 10^6 Å) and a Waters 410 differential refractometer, with a flow rate of 1.0 mL min^{-1} (in THF) and calibrated with polystyrene standards.

The monomers **2d** and **2f** were prepared according to literature procedures.[1,2] Below are adapted procedures from these references.

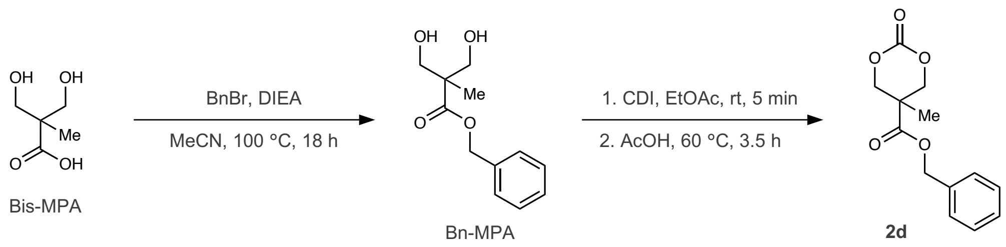

Synthesis of benzyl 3-hydroxy-2-(hydroxymethyl)-2-methylpropanoate (Bn-MPA).[1] A round-bottom flask equipped with a magnetic stir-bar was charged with 2,2-bis(hydroxymethyl) propionic acid (5.0 g, 37.3 mmol), DIEA (6.42 mL, 37.3 mmol) and MeCN (60 mL). The reaction mixture was stirred until it had become homogenous (~5 min) and BnBr (4.03 mL, 33.9 mmol) was added. The round-bottom flask was equipped with a reflux condenser and heated to 100 °C until the benzyl bromide had been completely consumed (18 h). Once complete, the reaction mixture was removed from the oil bath and allowed to cool to rt. The solvent was then removed with the aid of the rotary evaporator and the crude residue was dissolved in EtOAc (50 mL) and poured into 1 M HCl (100 mL). The phases were separated, and the aqueous layer was extracted twice more with EtOAc (50 mL). The combined organic layers were dried over Na_2SO_4, filtered, and concentrated with the aid of a rotary evaporator. The crude material was further purified by recrystallization from EtOAc/hexanes to afford the desired product as a white crystalline solid (5.89 g, 77%).

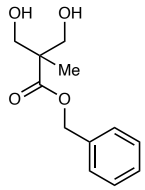

Synthesis of 2d.[1] A round-bottom flask equipped with a magnetic stir-bar was charged with the Bn-MPA (2.0 g, 8.9 mmol), MeCN (40 mL), and the reaction mixture was stirred at rt until the diol had fully dissolved. CDI (2.19 g, 13.5 mmol) was added and the reaction was stirred for an additional 10 min. AcOH (8.2 mL, 144 mmol) was added to the reaction mixture. The reaction mixture was equipped with a reflux condenser and heated to 75 °C for 3.5 h in a pre-heated oil bath. After 3.5 h, the reaction mixture was removed from the oil bath, cooled to room temperature, and then concentrated with the aid of a rotary evaporator. The crude residue was dissolved in EtOAc (100 mL) and poured into 2M HCl (100 mL). The organic and aqueous phases were separated, and the aqueous phase was extracted further with EtOAc (2 x 100 mL). The combined organic layers were dried over $MgSO_4$, filtered, and concentrated. The residue was dissolved in a solution of EtOAc (50 mL) and PhMe (50 mL) and concentrated using a rotary evaporator to remove the AcOH. The crude material was transferred to a 250 mL Erlenmeyer flask and dissolved in a minimal amount of THF (~8 mL). The solution was diluted with 125 mL MTBE and hexanes (~50 mL) was added with agitation until crystals had formed. The slurry was cooled to –20 °C in a freezer overnight. The slurry was then filtered, and the filter cake was washed with cold Et_2O (2 x 20 mL) and dried to give the desired product as white crystals (1.73 g, 78%).

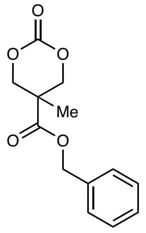

Synthesis of 2f.[2] A Schlenk tube (250 mL), equipped with a stir-bar and an addition funnel (250 mL), was charged with TsCl (4.4 g, 23.0 mmol), TMEDA (0.90 g, 7.50 mmol), Et_3N (5.3 g, 23.0 mmol), and MeCN (80 mL). A solution of 2,2'-(4-methylphenylimino)diethanol (3.0 g, 15.0 mmol) dissolved in MeCN (80 mL) was added to the addition funnel. The reaction flask was cooled to 0 °C in an ice bath and the atmosphere was purged with CO_2 for 5 min. Afterwards, the apparatus was sealed, and the diol solution was added dropwise to the solution over 15 min. After 1.5 hours, the reaction mixture was filtered to remove solids and the filtrate was concentrated with the aid of a rotary evaporator. The isolated crude material was dissolved in a minimal amount of $CHCl_3$ and purified by filteration through a plug of silica gel, eluting with 25% EtOAc in hexanes. Recrystallization from EtOAc/hexanes afforded the title compound as a white crystalline solid (2.85 g, 85%).

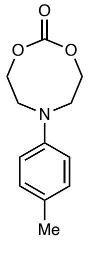

The catalyst **4c** was prepared according to a literature protocol,[3] below is an adapted procedure from this reference.

Synthesis of 4c.[3] To a 100 mL round-bottom flask equipped with a magnetic stir-bar, benzoyl chloride (1.55 g, 11.04 mmol), Et_3N (1.17 g, 11.54 mmol), and THF (20.1 mL) were added. 3,5-bis(trifluoromethyl)aniline (2.30 g, 10.04 mmol) was added dropwise over 5 min. After stirring at rt for 4 h, the reaction mixture was diluted with EtOAc, transferred to a separatory funnel, and washed three times with 1 M HCl and one time with brine. The organic layer was dried over $MgSO_4$, filtered, and concentrated with the aid of the rotary evaporator. The obtained solids were purified by flash column chromatography on silica gel (gradient heptane/EtOAc) to afford the title compound as a white solid (2.71 g, 81 %).

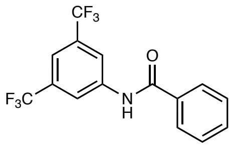

Characterization data for the title compound were in agreement with published data.[4]

The catalyst **5a** was prepared according to a literature protocol,[5] below is an adapted procedure from this reference.

Synthesis of 5a.[5] To a 100 mL round bottom flask equipped with a stir-bar, 3,5-bis(trifluoromethyl)phenyl isothiocyanate (2.10 g, 9.67 mmol) and THF (19.3 mL) were added. 2-aminopyridine (0.96 g, 10.15 mmol) was added and the reaction mixture was allowed to stir at rt for 15 h. The THF was removed with the aid of a rotary evaporator and the crude product purified by precipitation from THF into methanol (three times). After isolating and drying the solids the title compound was obtained as a white solid (2.62 g, 87 %).

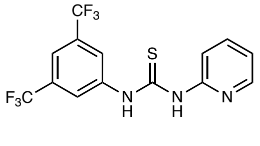

Synthesis of 5c. To a 100 mL round bottom flask, 2-chloro-1,3-dimethylimidazolinium chloride (3.00 g, 17.0 mmol), cyclohexylamine (1.76 g, 17.0 mmol) and sodium phosphate tribasic (4.45 g, 21.0 mmol) were dissolved in chloroform (45 mL) and heated to 50 °C for 20 h in an oil bath. After 20 h, the reaction removed from the oil bath and allowed to cool before filtering. The filtrate was concentrated with the aid of rotary evaporator. The isolated crude residue was dispersed in PhMe (40 mL) and 2 M NaOH (40 mL) was added, and the mixture was stirred for 1 h. The mixture was then transferred to a separatory funnel with additional toluene (80 mL) and 2 M NaOH (200 mL) and the aqueous and organic layers were separated. The organic layer was dried over $NaSO_4$, filtered, and concentrated with the aid of the rotary evaporator to afford the title compound as an oil (1.86 g, 56%).

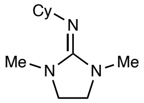

^{1}H NMR (400 MHz, $CDCl_3$): δ (ppm) 7.21 (m, 2H), 7.16 (m, 2H), 5.16 (m, 6H), 5.05–5.30 (s, overlapped, 2H), 2.35 (s, 3H), 1.58 (m, 3H).
^{13}C NMR (100 MHz, $CDCl_3$): δ (ppm) 154.5, 54.6, 37.7, 27.0, 25.9.

Synthesis of 5d. To a 100 mL round bottom flask, 2-chloro-1,3-dimethylimidazolinium chloride (2.00 g, 11.0 mmol), benzyl amine (2.50 g, 23 mmol) and sodium phosphate tribasic (3.00 g, 14 mmol) was heated to 50 °C for 20 h. After 20 h, the reaction removed from the oil bath and allowed to cool before filtering. The filtrate was concentrated with the aid of rotary evaporator. The isolated crude residue was dispersed in PhMe

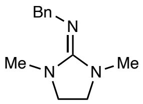

(40 mL) and 2 M NaOH (40 mL) was added and allowed to stir for 1 h. The reaction was transferred to a separatory funnel with vigorous shaking. The organic layer was dried over $NaSO_4$, filtered, and concentrated with the aid of the rotary evaporator to afford the title compound as an oil (0.95 g, 42%).

^{1}H NMR (400 MHz, $CDCl_3$): δ (ppm) 7.68 (m, 2H), 7.31 (m, 2H), 7.15, (m, 1H), 4.80 (m, 2H), 2.75–2.35 (br m, 10H).
^{13}C NMR (100 MHz, $CDCl_3$): δ (ppm) 157.35, 145.1, 128.9, 127.9, 126.51, 51.7, 47.0, 45.0, 31.2.

Characterization data for the title compound were in agreement with published data.[6]

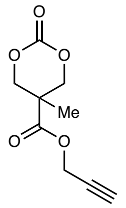

Synthesis of 6d. To a 100 mL round bottom flask equipped with a stir bar, propargyl bromide (80 wt. % in toluene) (4.90 g, 41.0 mmol), bis(hydroxy) propionic acid (5.00 g, 37.0 mmol), and DIEA (4.77 g, 37.0 mmol), and MeCN (50 mL) were added. The flask was equipped with a condenser and heated to 60 °C in an oil bath for 20 h. The reaction was then cooled to room temperature and transferred to a 250 mL flask containing an additional 100 mL of MeCN. To this solution, CDI (19.9 g, 0.123 mol) was added over a 5 min and once dissolved, AcOH (18.8 mL, 0.328 mol) was added in increments over a 5 min. The reaction was heated to 80 °C for 2.5 h. The reaction mixture was cooled, concentrated with the aid of a rotary evaporator, and dissolved in EtOAc (150 mL). The solution was transferred to a separatory funnel and washed 3 times with 1.5 M HCl followed by a wash with brine. The organic layer was dried over $MgSO_4$ followed by the addition of 40 mL of PhMe and concentration using a rotary evaporator. Additional PhMe (40 mL) was added and concentrated to remove remaining acetic acid. This process was repeated 3 times to yield the title compound as a clear oil that crystallized overnight (1.65 g, 22% over two steps). The monomer was used without further purification.

^{1}H NMR (400 MHz, $CDCl_3$): δ (ppm): 4.80 (s, 2H), 4.74–4.71 (d, J=10.8 Hz, 2H), 4.23–4.21 (d, J=10.8 Hz, 2H), 2.5 (s, 1H), 1.37 (s, 3H).

Characterization data for the title compound were in agreement with published data.[7]

Supplementary Discussion

CMDL is a domain specific language developed to enable an extensible approach for experimental documentation while leveraging features of modern integrated development environments (IDE) to assist in the documentation process. It should be noted that the language and syntax features described below reflect the version used in the paper. For the most up to date documentation and examples, please see the GitHub repository and the associated documentation webpage. In depth tutorials on CMDL and the IBM Materials Notebook can be found at the GitHub repository along with numerous example CMDL notebook documents. Below is an abbreviated introduction to CMDL within the IBM Materials notebook environment.

CMDL Introduction. CMDL has two primary syntactical features—*groups* and *properties*. *Groups* may contain any number of different *properties* as well as nested sub-*groups*. *Properties* contain

key–value pairs describing different data types and are always enclosed within a *group* (Supplementary Fig. 1).

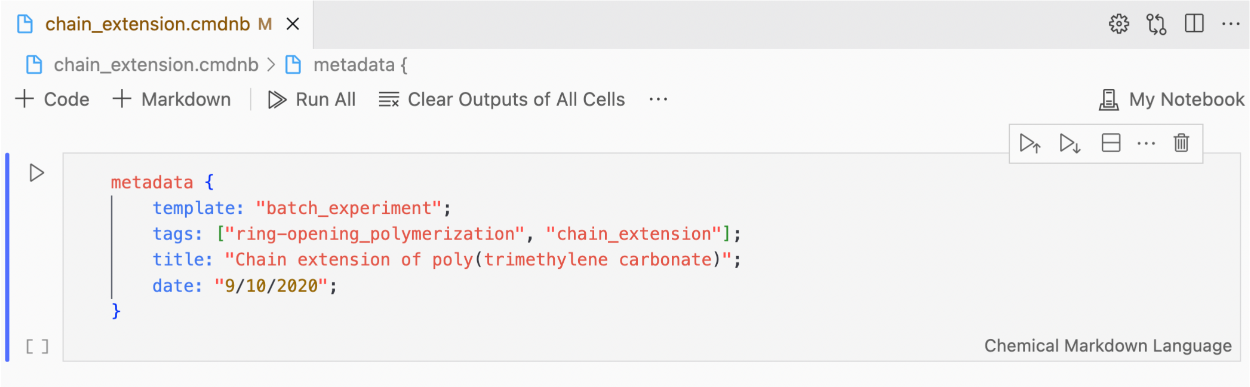

Supplementary Fig. 1. Example *metadata* group in CMDL. Screenshot from of a *metadata* group contains several key–value pairs describing different properties. All the *properties* (*template, title, date*) except for *tags* have string values as indicated by the enclosing double quotes. The *tags* property has multiple string values as list format enclosed by square brackets.

The number and type of properties that may allowed within a *group* or allowable ranges for data for a particular property are defined and enforced by the CMDL compiler. As noted in the manuscript, the term compiler is used loosely as this portion of the program performs static analysis of the CMDL text. Erroneous values and invalid syntax are immediately flagged by the compiler and are highlighted (Supplementary Figs. 2–3).

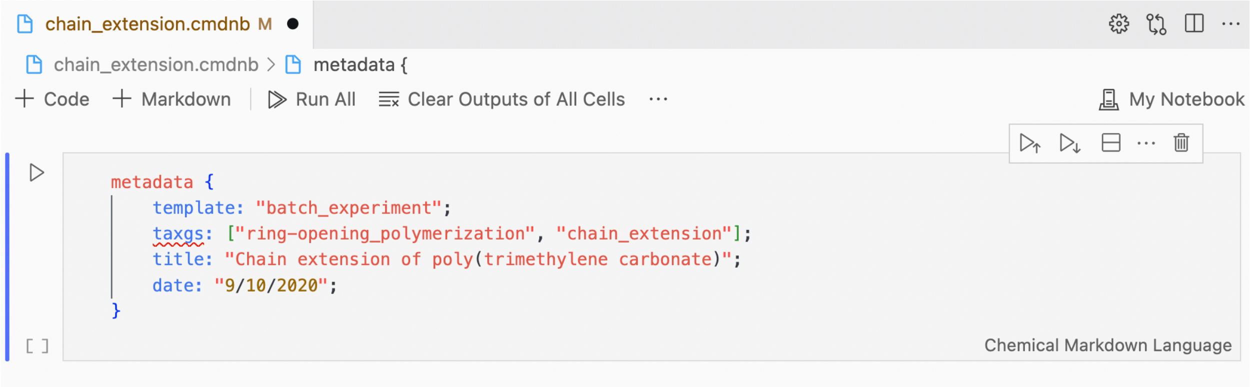

Supplementary Fig. 2. Error highlighting for misspelled property. Screenshot of error diagnostics highlighting an invalid property on the *metadata* group.

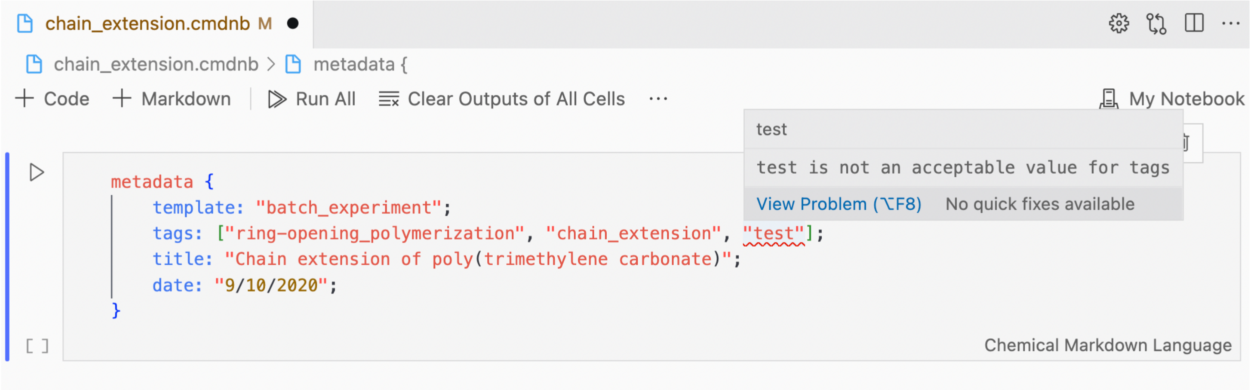

Supplementary Fig. 3. Error highlighting for an invalid value for a *property*. Screenshot of an error diagnostic highlighting an invalid value on the *tags* property. Highlighting the erroneous value produces the popup window describing the error.

Groups can come in several different types. Supplementary Figure 1 depicts a generic *group* describing the metadata for a particular experiment in a notebook document. Other *group* types include a *named group*, which can be used to define *references* in CMDL. *References* are groups that describe a particular entity within an experiment, such as a chemical, polymer, or continuous-flow reactor, and allow data for that entity to be used in multiple locations within the record without redefinition. Supplementary Figure 4 shows example references, including an example of a reference which was imported.

```
import THF from "cmdl.lib";

chemical kOtBu {
    molecular_weight: 112.212 g/mol;
    state: "solid";
    smiles: "[K+].[O-]C(C)(C)C";
}

chemical TMC {
    molecular_weight: 102.3 g/mol;
    state: "solid";
    smiles: "O=C1OCCCO1";
}
```

✓ 0.1s Chemical Markdown Language

Supplementary Fig. 4. Example named groups in CMDL. Screenshot of *named groups* (chemical) describing *references* for potassium *tert*-butoxide (*kOtBu*) and trimethylene carbonate (*TMC*).

These entities may be used in more than one place within a single experiment record. For instance, a solvent such as Ethyl Acetate may be used both in the reaction itself and as the organic phase in a biphasic extraction during workup. Using a reference and appending additional data is accomplished by the "@" prefix. Supplementary Fig. 5 shows an example of a *named group* (reaction) containing references to chemicals or polymers being used in the reaction.

```
reaction ChainExtension {
    temperature: 22 degC;

    @MeO-pTMC20 {
        mass: 20 mg;
        roles: ["initiator"];
    };

    @kOtBu {
        mass: 2 mg;
        roles: ["catalyst"];
    };

    @TMC {
        mass: 200 mg;
        roles: ["monomer"];
    };

    @THF {
        volume: 2 ml;
        roles: ["solvent"];
    };

    @MeO-pTMC200 {
        roles: ["product"];
    };
}
```

Chemical Markdown Language

Supplementary Fig. 5. Example reaction group. Screenshot of reaction group using references to chemicals defined elsewhere (Supplementary Fig. 4) to describe a chemical reaction.

By using the different *group* types and their associated *properties*, we can define a highly extensible data model as new *properties* can easily be added to existing groups as well as be re-used across multiple different *groups*, all with the compiler enforcing type checks. Once valid CMDL is written and checked by the compiler, it can be executed using the kernel which is part of the IBM Materials Notebook. During execution, the CMDL interpreter will perform some basic calculations based on the group type and its associated model. For example, the interpreter will compute basic stoichiometry and estimate concentrations during execution of a reaction group (Supplementary Fig. 6). The output is by default rendered as JSON, however it can be displayed as a table using the custom notebook renderer.

```
        roles: ["solvent"];
    };

    @MeO-pTMC200 {
        roles: ["product"];
    };
}
```

[7] ✓ 0.1s Chemical Markdown Language

ChainExtension

Temperature: 22 ° C

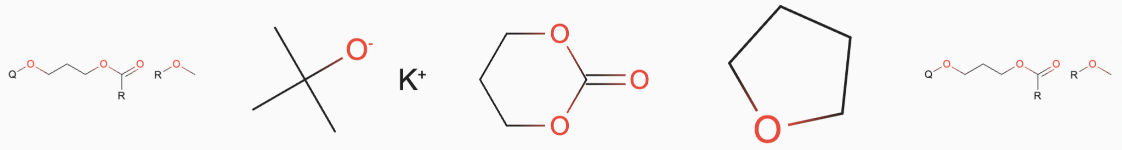

Name	Mw	Density	Mass	Volume	Moles	Ratio	Roles	Molarity	Molality	Mols/Vol
MeO-pTMC20	2000	—	20 mg	—	0.01 mmol	1	initiator	0.0045 mol/l	0.006964 mol/kg	0.005 mol/l
kOtBu	112.212	—	2 mg	—	0.0178 mmol	1.78	catalyst	0.008021 mol/l	0.0124 mol/kg	0.008912 mol/l
TMC	102.3	—	200 mg	—	1.955 mmol	195.5	monomer	0.8799 mol/l	1.3614 mol/kg	0.9775 mol/l
THF	123.12	0.718	1.436 g	2 ml	0.0117 mol	1166.34	solvent	5.2491 mol/l	8.1222 mol/kg	5.8317 mol/l

+ Code + Markdown

Supplementary Fig. 6. Example reaction output. Screenshot of reaction group output after running the cell. The CMDL interpreter reads the valid CMDL syntax and performs a stoichiometry calculation for the reaction based on available data. The data for each of chemicals, either defined using CMDL elsewhere or imported (Fig. S4) is merged with the values defined for them within the *reaction* group for the calculation.

CMDL Polymer Graphs. CMDL comes with built-in support for defining polymer graphs. The CMDL syntax for polymer graphs is composed of three elements, the top-level *polymer graph* group itself, *containers*, and *connections*. Additionally, discrete structural elements are defined separately as *fragments*, and referenced within the *polymer graph* definition. This allows definition of polymer graphs using CMDL as a composite tree. Each *container* (including the *polymer graph* group) may define which discrete nodes exist within them. Additionally, each *container* or *polymer graph* will define *connection* properties (defined by angle brackets)—representing edges in the polymer graph—for only nodes defined within them or nested *container* groups. *Fragments* can potentially be referenced in multiple locations within a polymer graph, depending on the polymer structure.

```
fragment eg_MeO {
    molecular_weight: 31.02 g/mol;
    smiles: "CO[R:1]";


    point R {
        quantity: 1;
    };

}
fragment p_TMC {
    molecular_weight: 102.3 g/mol;
    smiles: "O=C([R:1])OCCCO[Q:2]";


    point R {
        quantity: 1;
    };


    point Q {
        quantity: 1;
    };

}
```

[2] ✓ 0.4s Chemical Markdown Language

...

eg_MeO

Molecular Weight: 31.02 g/mol

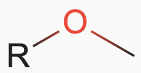

p_TMC

Molecular Weight: 102.3 g/mol

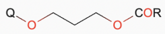

Supplementary Fig. 7. Example fragments for use within a polymer graph definition. Screenshot of fragment groups for defining discrete structural elements within a polymer graph definition. The *point* groups defined on each fragment enable the CMDL compiler to recognize specific attachment points on each fragment for creating connection objects.

Supplementary Figure 8 depicts the CMDL syntax for a simple poly(trimethylene carbonate) homopolymer initiated from methanol. The *fragments* which define the methanol initiator (eg_MeO) and the trimethylene carbonate repeat unit (p_TMC) are defined separately (Supplementary Fig. 7) and referenced within the polymer graph (Supplementary Fig. 8). On each *fragment* a *point* group is defined for each distinct attachment point within the SMILES string, allowing these points to be referenced within connection properties (Supplementary Fig. 7). The *polymer graph* group itself contains a reference to the methanol *fragment* in the *nodes* property. The *polymer graph* group also has a connection *property* (defined with angle brackets) to define an edge connection between the methanol node and the trimethylene carbonate repeat unit.

```
polymer_graph TMC-Graph {
    nodes: [ @eg_MeO ];
    <@eg_MeO.R => @TMC_Block.p_TMC.R>;

    container TMC_Block {
        nodes: [ @p_TMC ];
        <@p_TMC.Q => @p_TMC.R>;

    };
}
```

[3] ✓ 0.1s Chemical Markdown Language

Supplementary Fig. 8. Example polymer graph for a carbonate homopolymer. Screenshot of polymer graph for a poly(trimethylene carbonate) homopolymer.

Nested within the *polymer graph* group is the *container* group for the trimethylene carbonate block, which references the trimethylene carbonate fragment within the nodes property and defines the self-referencing connection for the trimethylene carbonate repeating connection (Supplementary Fig. 8). By convention, each *polymer graph* group or *container* group may define connections between its own nodes and between its nodes and those of nested containers. Repeating units, such as trimethylene carbonate in the case of Supplementary Figure 8, are typically separated into their own containers as it allows clear delineation of repeating structures and discrete end groups or other structural moieties. This is especially convenient in the case of multiblock architectures, statistical copolymers, grafted polymers, or more complex polymer architectures.

```
polymer_graph BASE {
    nodes: [ @eg_PyreneBuOH ];
    <@eg_PyreneBuOH.R => @Valerlactone_Block.p_VL.R>;
    <@Valerlactone_Block.p_VL.Q => @Lactide_Block.p_raclac.R>;

    container Lactide_Block {
        nodes: [ @p_raclac ];
        <@p_raclac.Q => @p_raclac.R>;
    };

    container Valerlactone_Block {
        nodes: [ @p_VL ];
        <@p_VL.Q => @p_VL.R>;
    };
}
                                                    Chemical Markdown Language
```

Supplementary Fig. 9. Example polymer graph for a block copolymer. Screenshot of polymer graph for a poly(valerolactone)-b-poly(L-lactide) block copolymer.

An AB block copolymer can be defined by adding a second nested *container* group and defining additional *connection* properties and *node* references. Supplementary Figure 9 shows an example poly(valerolactone)-b-poly(L-lactide) block copolymer initiated from pyrenebutanol.

```
polymer_graph BASE {
    nodes: [ @eg_PyreneBuOH ];
    <@eg_PyreneBuOH.R => @Carbonate_Block.p_TMC.R | @Carbonate_Block.p_TMCPrCl.R>;

    container Carbonate_Block {
        nodes: [ @p_TMC, @p_TMCPrCl ];
        <@p_TMC.Q | @p_TMCPrCl.Q => @p_TMC.R | @p_TMCPrCl.R>;

    };
}
                                                    Chemical Markdown Language
```

Supplementary Fig. 10. Example polymer graph for a statistical homopolymer. Screenshot of polymer graph for a carbonate statistical copolymer.

A statistical copolymer is readily defined when two (or more) repeat units are defined on the same *container* group. Additionally, *connection* properties would have to be added for each connection between the different repeat units and each repeat unit with itself. This is somewhat tedious, so instead we can use a syntactical shortcut with the pipe ("|") and express the distributed connections between repeat units in a statistical copolymer (Supplementary Fig. 10).

Consuming polymer graphs. The definition of a *polymer graph* using CMDL syntax simply defines the base structural features and connectivity of a polymeric material. The graph definition can then be consumed within the definition of a polymer *reference*.

```
polymer MeO-pTMC20 {
    tree: @TMC-Graph;
    mn_avg: 2000 g/mol;
    dispersity: 1.12;
    state: "solid";

    @TMC-Graph.TMC_Block.p_TMC {
        degree_poly: 20;
    };
}

polymer MeO-pTMC200 {
    tree: @TMC-Graph;
    state: "solid";
}
```

[4] ✓ 0.4s Chemical Markdown Language

MeO-pTMC20 MeO-pTMC200

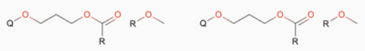

Supplementary Fig. 11. Example of a definition of polymer reference. Screenshot of polymer reference definition (*MeO-pTMC20* and *MeO-pTMC200*). The *tree* property references the polymer graph definition.

In Supplementary Figure 11, the polymer references for a poly(trimethylene carbonate) polymers are defined, one for the known starting material (*MeO-pTMC20*) and one for the new, chain extended product (*MeO-pTMC200*).The DP_n is assigned to the poly(trimethylene carbonate) repeat unit in the starting material for the chain extension reaction. Following the experiment, the DP_n value for the product polymer (*MeO-pTMC200*) can be assigned based on measured values in the *sample* group (Supplementary Fig. 12).

```
sample CE-I-1 {
    time_point: 200 s;

    nmr CE-I-1A {
        nmr_nuclei: "1H";

        @TMC {
            conversion: 93%;
        };

        @MeO-pTMC200.TMC_Block.p_TMC {
            degree_poly: 200;
        };

        @MeO-pTMC200 {
            mn_avg: 20001 g/mol;
        };
    };

    gpc CE-I-1B {
        @MeO-pTMC200 {
            mn_avg: 14000 g/mol;
        };
    };
}
```

[7] ✓ 0.1s Chemical Markdown Language

Supplementary Fig. 12. Example of a sample group and nested characterization groups. Screenshot of a sample group describing two characterization experiments (*nmr* and *gpc*). Measured values from these experiments are attached to the referenced chemical and polymer entities.

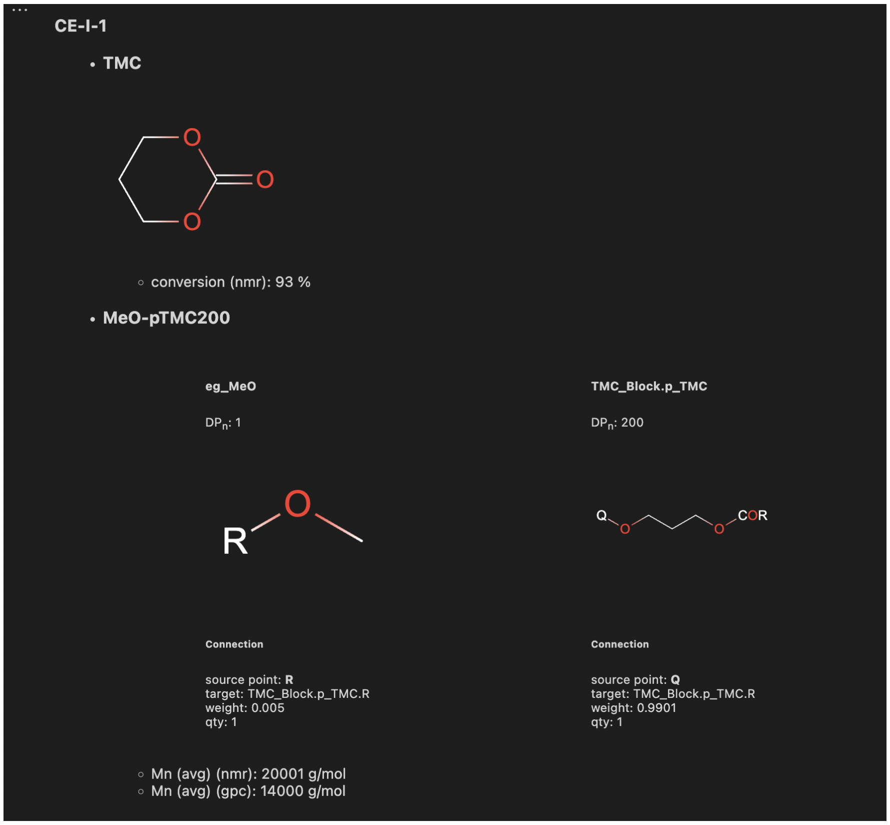

Supplementary Fig. 13. Example of the cell output of the sample group from Supplementary Figure 12. Screenshot of a sample group output summarizing the results from the sample. Weights for the polymer fragments are computed based on labeled DP_n values.

Continuous-flow experiments using CMDL. Similar to *polymer graphs*, graphs representing continuous-flow reactors can be defined and consumed within continuous-flow reactions. Each *component* group defined in a reactor represents a physical piece of hardware, such as pumps, stock solution tanks, or reactor tubing. Reactor graphs are directed, and the *target* property of the *component* group defines the edge between nodes as well as the direction of flow.

```
reactor_graph FlowTest {
    component MonomerTank {
        description: "Tank for monomer solution";
        target: @PolyReactor.Mixer;
    };

    component CatalystTank {
        description: "Tank for catalyst solution";
        target: @PolyReactor.Mixer;
    };

    reactor PolyReactor {
        component ReactorTube {
            target: @FlowTest.Collection;
            volume: 2 ml;
            inner_diameter: 0.1 mm;
        };

        component Mixer {
            description: "PEEK T-mixer";
            target: @ReactorTube;
        };

    };

    component Collection {
        description: "reactor output";
    };
}
```

[2] ✓ 0.1s Chemical Markdown Language

Supplementary Fig. 14. Example of reactor graph definition. Screenshot of reactor graph defining individual *components* and *reactors*.

Components may also be grouped within a *reactor* group. Any component defined under a *reactor* group will be considered as part of a single reactor and contribute to its total reactor volume. Reaction stoichiometry and estimated residence times will be computed for each *reactor* group defined within the reactor graph. Supplementary Fig. 14 shows the definition of a reactor group (PolyReactor) using CMDL syntax.

Stock solutions for each input node on the reactor graph are also defined in separate groups, similar to how *reaction* groups are defined (Supplementary Fig. 14). Upon execution of a cell containing a *stock solution* group, the stoichiometry is computed by the CMDL interpreter and displayed in a table in the cell output.

```
solution CatalystSolution {
    @pMeBnOH {
        mass: 49.8 mg;
        roles: ["initiator"];
    };

    @kOtBu {
        mass: 11.2 mg;
        roles: ["catalyst"];
    };

    @THF {
        volume: 10 ml;
        roles: ["solvent"];
    };
}
```

[11] ✓ 0.9s Chemical Markdown Language

...

CatalystSolution

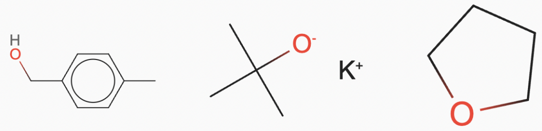

pMeBnOH kOtBu THF

Name	Mw	Density	Mass	Volume	Moles	Ratio	Roles	Molarity	Molality	Mols/Vol
pMeBnOH	122.16	—	49.8 mg	—	0.4077 mmol	4.08	initiator	0.0405 mol/l	0.0568 mol/kg	0.0408 mol/l
kOtBu	112.212	—	11.2 mg	—	0.0998 mmol	1	catalyst	0.009921 mol/l	0.0139 mol/kg	0.009981 mol/l
THF	123.12	0.718	7.18 g	10 ml	0.0583 mol	584.27	solvent	5.7964 mol/l	8.1222 mol/kg	5.8317 mol/l

Supplementary Fig. 15. Example of stock solution group. Screenshot of stock solution group, its chemical components, and the output after running the cell.

Once the reactor graph and stock solutions have been defined, they may be referenced within the *flow reaction* group. The reactor is referenced in the *reactor* property on the flow reaction group, whereas the stock solutions are referenced as *reference* groups (Supplementary Fig. 16). Within each stock solution *reference* group, the *input* property is defined and references the input node on the reactor graph. The *flow rate* property defines the flow rate for a stock solution input.

```
flow_reaction RunA {
    reactor: @FlowTest;

    @MonomerSolution {
        input: @FlowTest.MonomerTank;
        flow_rate: 10 ml/min;
    };

    @CatalystSolution {
        input: @FlowTest.CatalystTank;
        flow_rate: 10 ml/min;
    };

    @pMeBnO-pL-lac {
        roles: ["product"];
    };
}
```

[ ] Chemical Markdown Language

Supplementary Fig. 16. Example of flow reaction group. Screenshot of flow reaction group, its stock solution components, and the polymer product (*pMeBnO-pL-lac*).

Upon execution of the cell with the defined *flow reaction* the CMDL interpreter will use the referenced reactor graph and propagate the stock solutions through the graph. When stock solutions are mixed in a reactor, the dilution ratios and stoichiometry for the reaction will be computed along with the estimated residence time. These values will be displayed in the cell output (Supplementary Fig. S17).

RunA

Products:

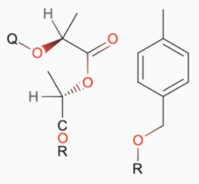

PolyReactor

Flow Rate: 20 ml/min

Volume: 2 ml

Residence Time: 6 s

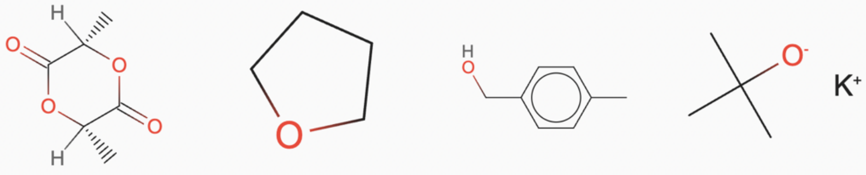

Name	Mw	Density	Mass	Volume	Moles	Ratio	Roles	Molarity	Molality	Mols/Vol
lactide	144.12	—	125.9658 mg	—	0.874 mmol	88.1	monomer	0.437 mol/l	0.6517 mol/kg	0.4679 mol/l
THF	123.12	0.718	1.3412 g	1.868 ml	10.8935 mmol	1098.07	solvent	5.4467 mol/l	8.1222 mol/kg	5.8317 mol/l
pMeBnOH	122.16	—	4.9498 mg	—	0.0405 mmol	4.08	initiator	0.0203 mol/l	0.0302 mol/kg	0.0217 mol/l
kOtBu	112.212	—	1.1132 mg	—	9.9206 mcmol	1	catalyst	0.00496 mol/l	0.007397 mol/kg	0.005311 mol/l

Supplementary Fig. 17. Example of flow reaction output. Screenshot of flow reaction group output from running the cell in Supplementary Fig. 16.

Compilation and export of experimental data. Once all the requisite data for a given experiment is recorded in the CMDL syntax and executed, saving the record will automatically create a JSON file using the default export schema (see the GitHub repository for examples). These JSON files may be loaded into a database or other AI pipeline as needed.

Inspection of generated polymer graphs. Data from the RT model for design of new polymers were inspected using the IBM Materials Notebook. This was accomplished by first cleaning the CSV output from the model and then serializing the data into CMDL syntax. The generated materials were then written to individual CMDL notebook files in groups of 50. It should be noted that during serialization a dummy molecular weight (123 g mol^{-1}) was given to new *fragment* groups, future work will aim to provide a more accurate molecular weight estimation for valid SMILES strings. While the CMDL compiler assisted in identifying erroneous syntax and missing components in the polymer graphs, future versions that assist in detection and/or correction of

invalid SMILES strings will be important to better enable experimentalists to inspect AI predictions. Nonetheless, the use of the IBM Materials Notebook to inspect the generated polymer structures proved invaluable. Supplementary Figure 18 depicts example serialized data and its output upon execution of the cell.

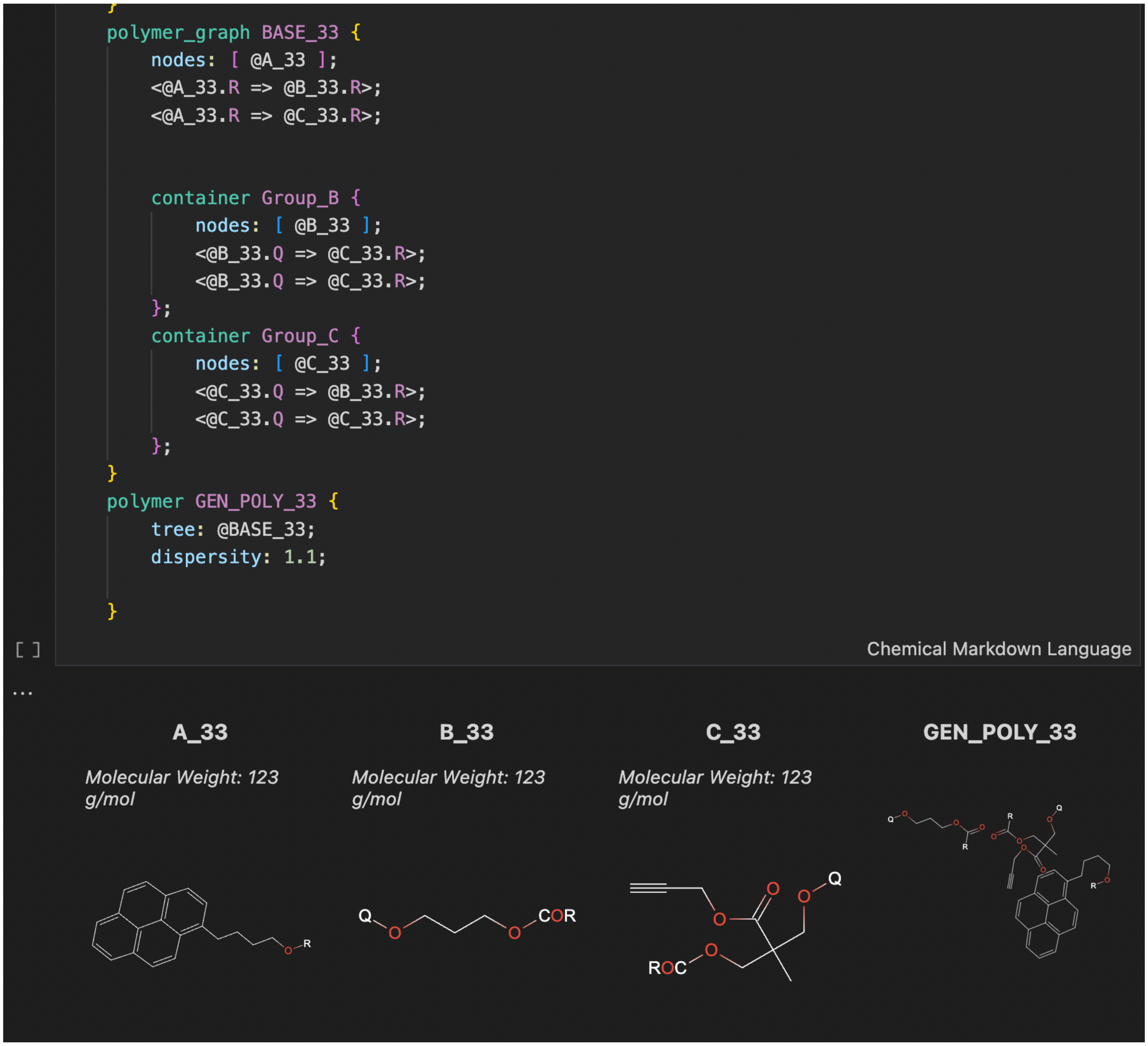

Supplementary Fig. 18. Example of a generated polymer serialized into CMDL. Screenshot of generated polymer (*Gen_Poly_33*), its polymer graph (*Base_33*), and a simple rendering of the polymer graph upon running the cell.

Supplementary Figures

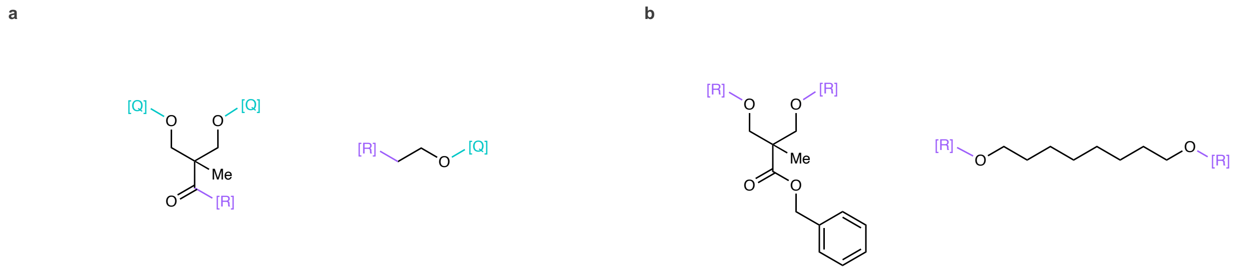

Supplementary Fig. 19. Examples of structures with symmetric and non-symmetric attachment points. a Examples of structures for polymer nodes with non-symmetric attachment points ([R] or [Q]) or more than a single type of attachment point based on chemical environment. Attachment points are differentiated by letter and color (blue or green). **b** Examples of structures for polymer nodes with more than one identical attachment point based on chemical environment or symmetry. Attachment points are highlighted in color.

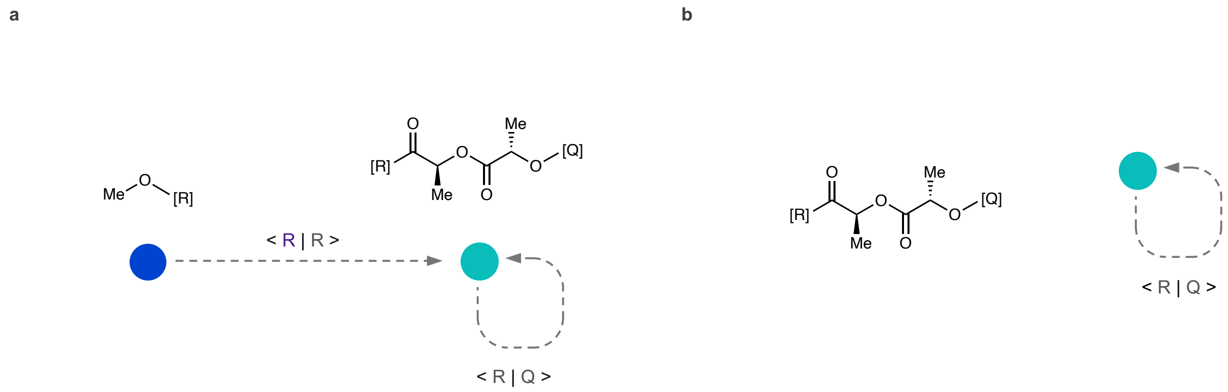

Supplementary Fig. 20. Examples of linear and cyclic polymer graph representations. a Example of a linear polymer graph containing a methoxy end group and a L-lactide repeat unit. Blue circle represents methoxy end group, dashed grey arrows are edges with the source and target attachment points (color coded by their respective nodes) in angle brackets (< source point | target point >). Green circle represents L-lactide repeat unit in the graph. **b** Example of a cyclic L-lactide polymer. Green circle and dashed grey arrow are the same as in **a**.

a

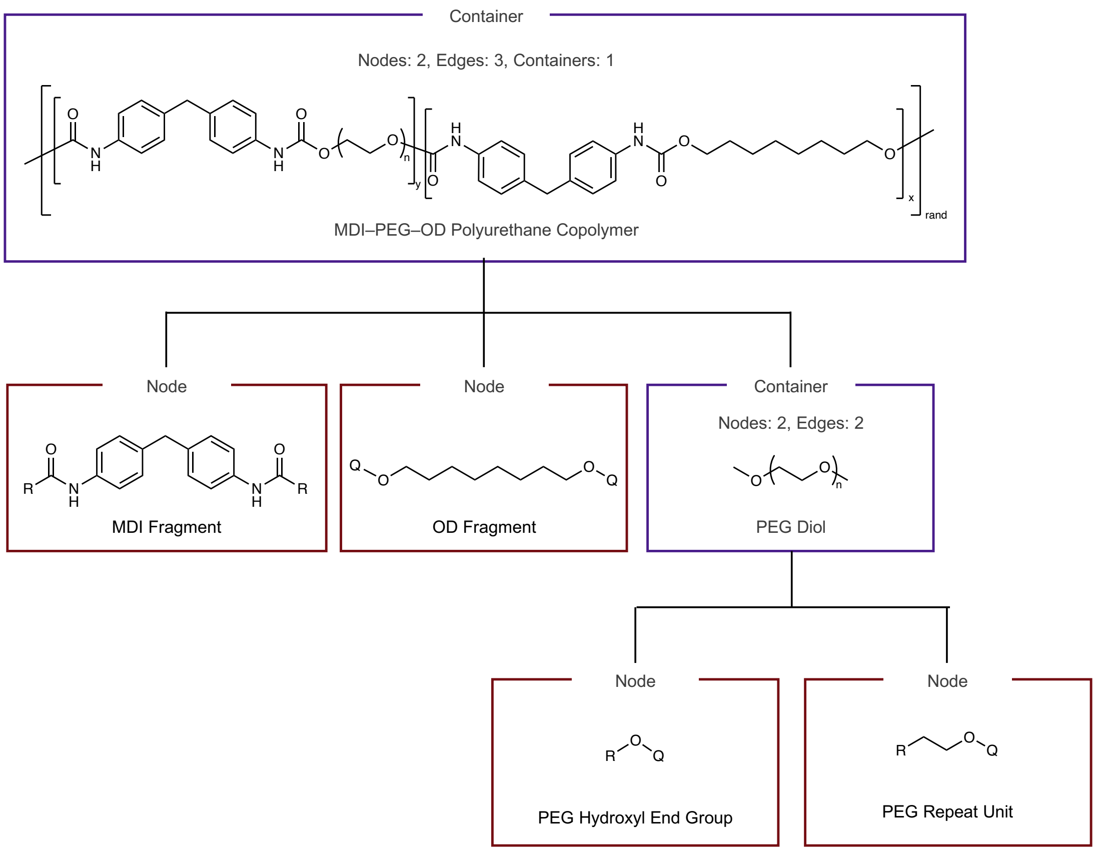

b

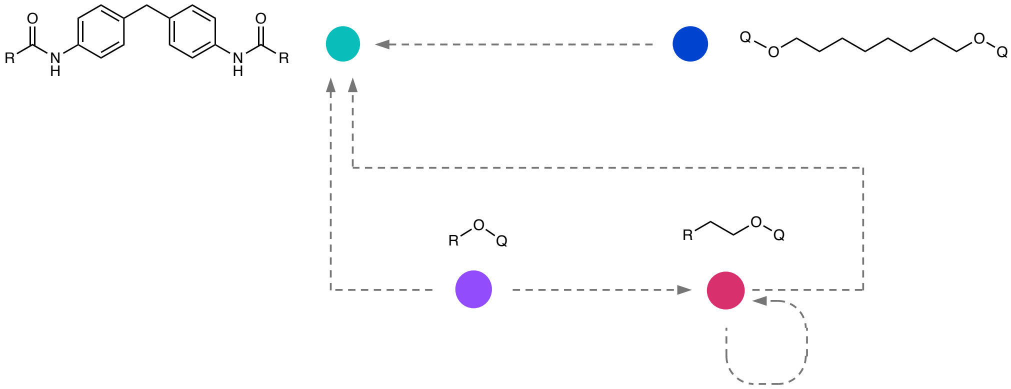

Supplementary Fig. 21. Examples of a composite tree and graph representation for a polyurethane copolymer. a Example of a composite tree for an MDI–PEG–OD polyurethane copolymer with connections between nodes omitted for simplicity. Each of the containers (blue outlined boxes) has either discrete fragments (MDI Fragment, OD Fragment, etc.) or other nested containers. Connections between elements are defined between fragments within a container or a nested container. For instance, a connection item between the PEG Repeat Unit and the MDI fragment would be within the MDI-PEG-OD Polyurethane Copolymer container and not on the PEG Diol container. **b** Corresponding graph representation of MDI-PEG-OD Polyurethane Copolymer. Edge definitions are omitted for simplicity.

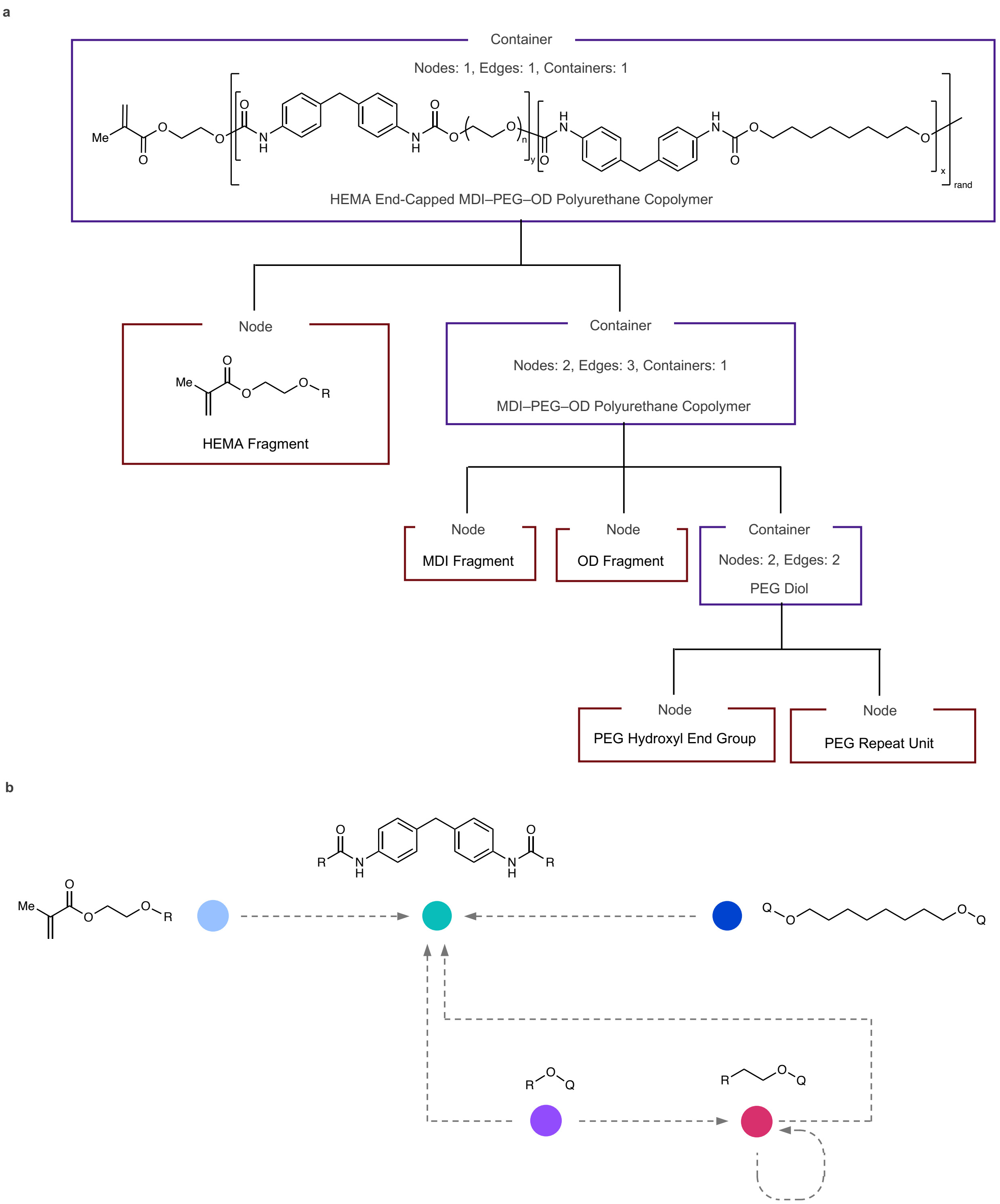

Supplementary Fig. 22. Examples of a composite tree and graph representation for an end-capped polyurethane copolymer. **a** Example of end-capped polyurethane copolymer. An additional container was added to the composite tree defined in Supplementary Fig. 21 to better define the connectivity of the end-group with the rest of the material. **b** Graph representation of the polyurethane copolymer. Edge definitions are omitted for simplicity.

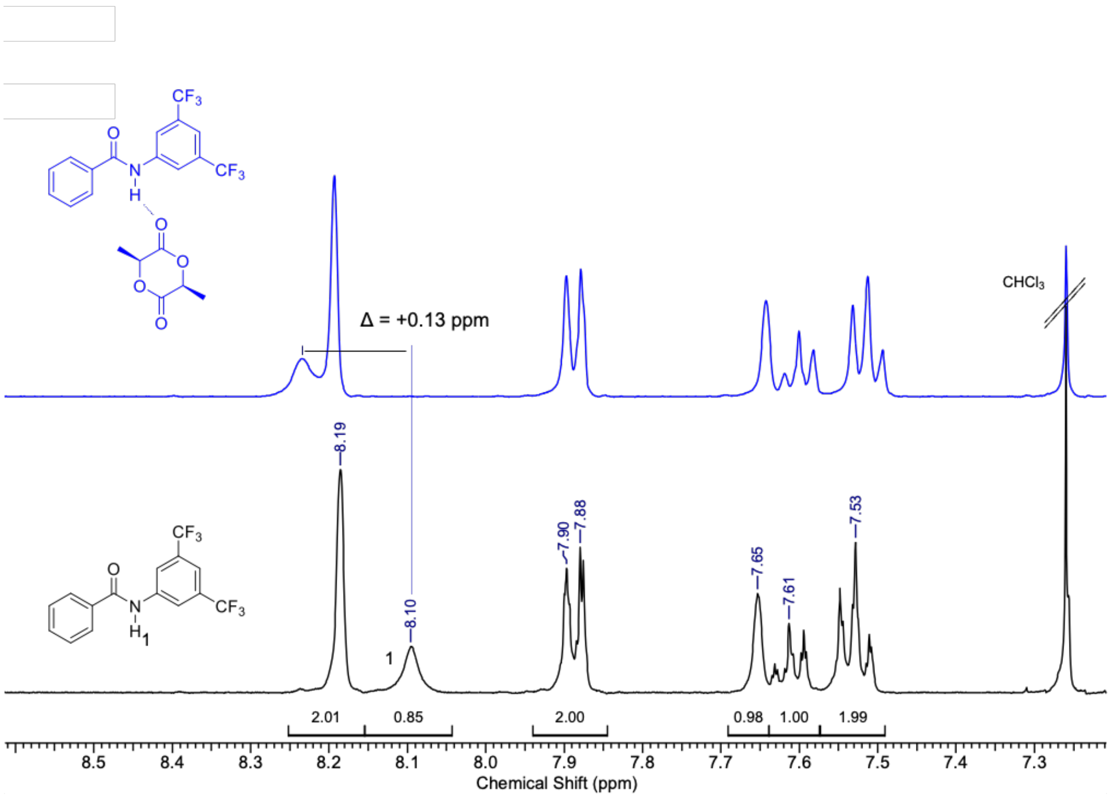

Supplementary Fig. 23. ^{1}H NMR Spectra in $CDCl_3$ showing activation of 2a with catalyst 4c. Top NMR spectrum in blue shows **4c** and **2a** mixture. Bottom NMR spectrum in black shows **4c** alone. N–H proton of **4c** is labeled in the spectrum with the number 1.

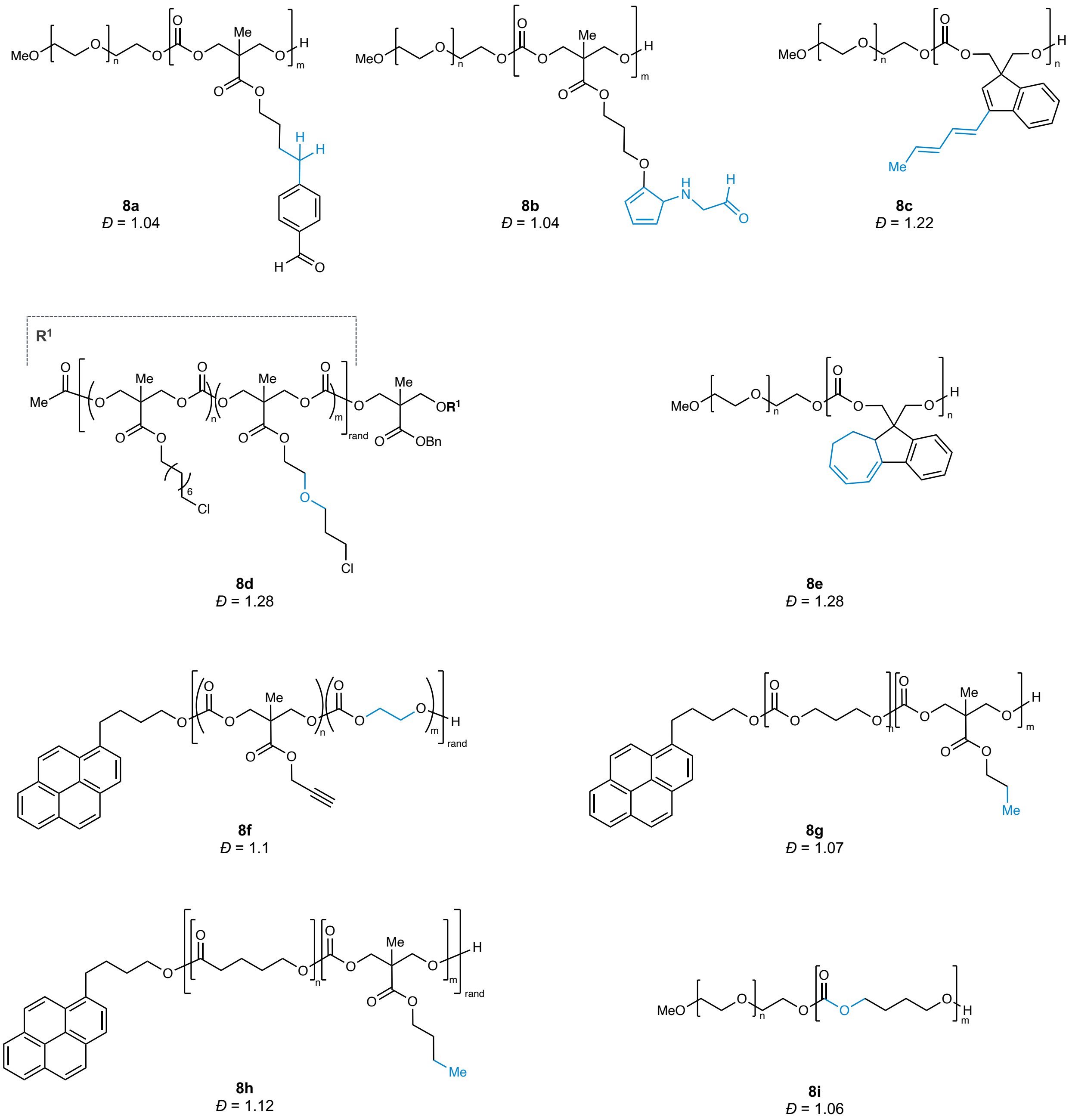

Supplementary Fig. 24. Examples of generated polymers with modified repeat units. Modified sections of the polymer structure, relative to seed data, are highlighted in color. These materials contain repeat units not part of the original training data.

a

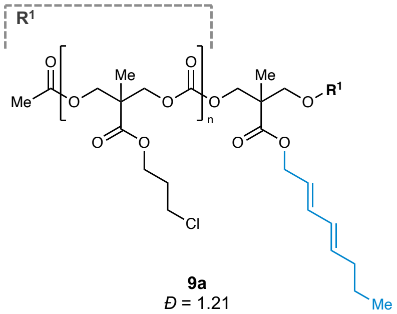

9a
$Đ$ = 1.21

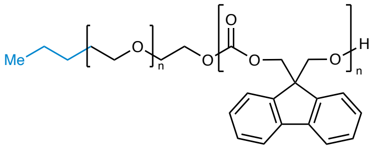

9b
$Đ$ = 1.19

b

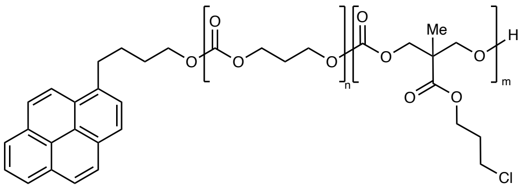

10a
$Đ$ = 1.07

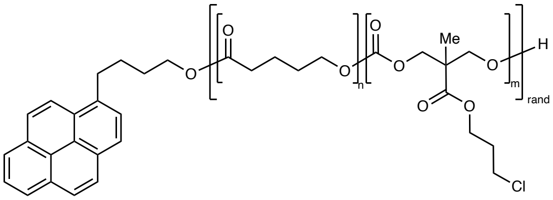

10b
$Đ$ = 1.07

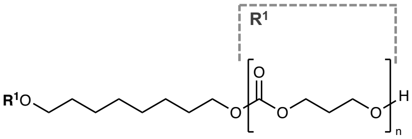

10c
$Đ$ = 1.09

Supplementary Fig. 25. Examples of generated polymers. a Examples of generated polymers with modified initiators. Modified sections are highlighted in color. **b** Examples of generated polymers with either new architectures or new monomer combinations not observed in training data.

a

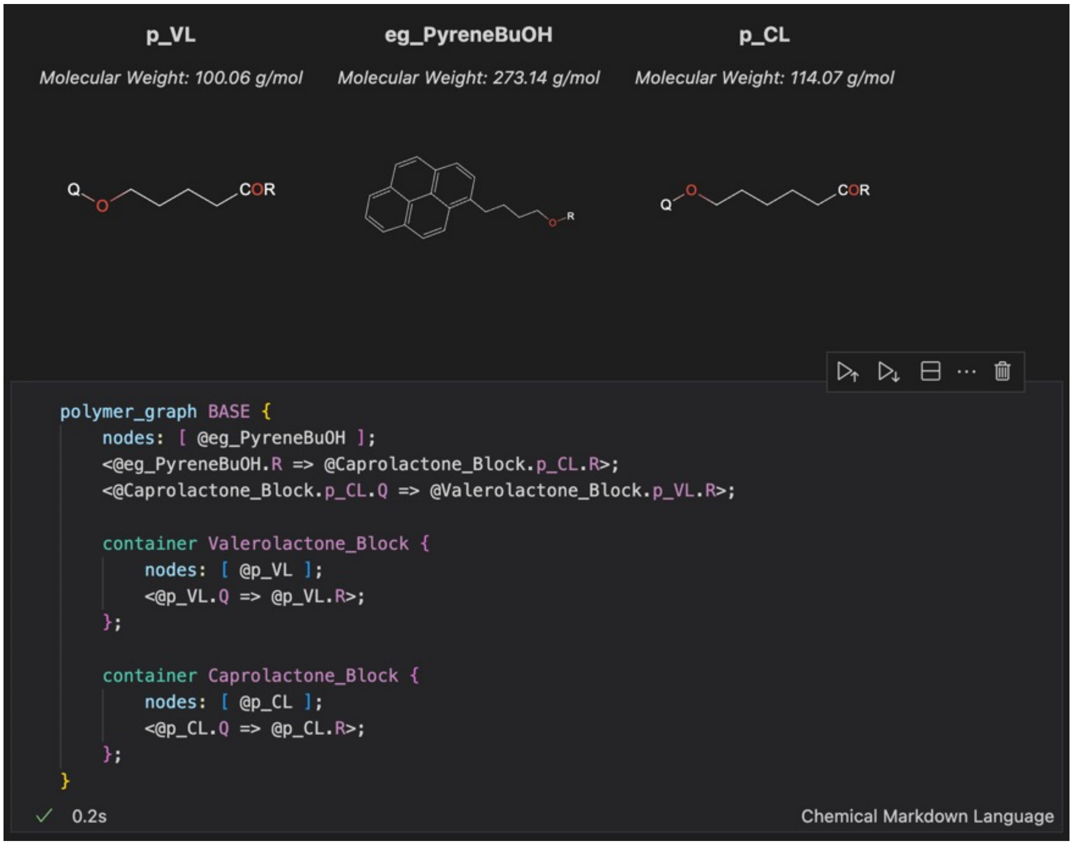

b

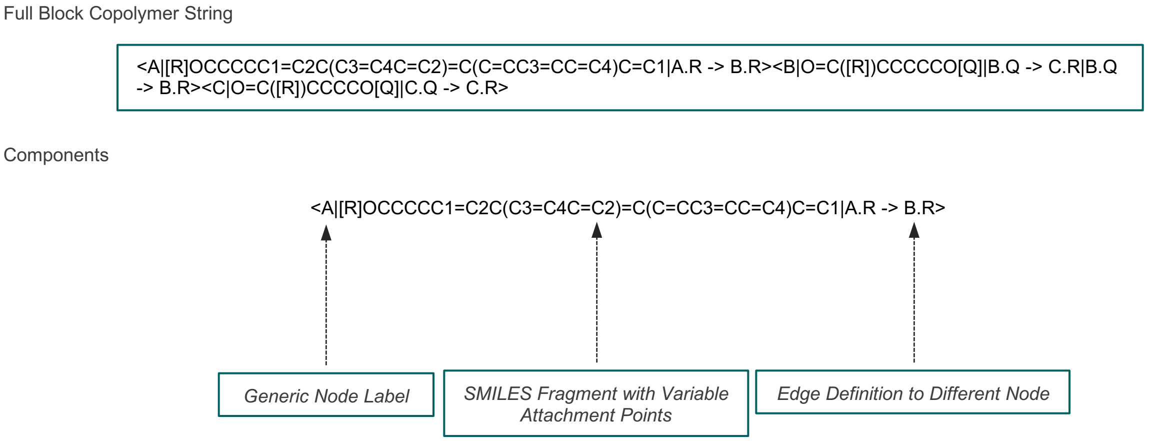

Supplementary Fig. 26. Example of conversion of graph representations to strings for use in RT models. a Screenshot of CMDL definition of a block copolymer. **b** Example of string representation output of the block copolymer polymer graph. Each node is enclosed with angle brackets wherein there is a generic label for a particular node, its SMILES string with variable attachment points, and edge definition(s). Each of these components is separated by a pipe ("|") character. If there are multiple edges originating from a node, each is also separated by a pipe character.

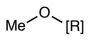

MeO

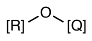

HO

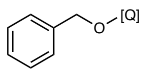

BnOH

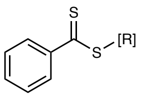

PhCSS

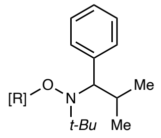

tBuPhiPrNO

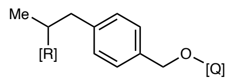

4BnOH

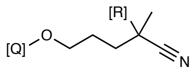

pentanolCN

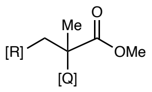

MMA

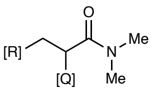

DMA

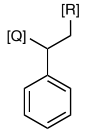

Styrene

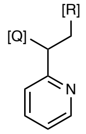

2VP

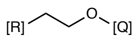

PEO

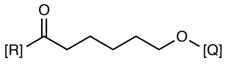

CL

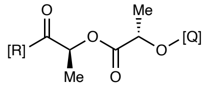

Llac

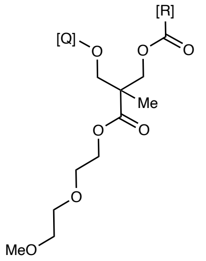

TMCDiEtGlyc

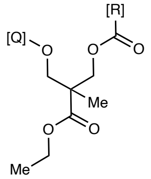

TMCEt

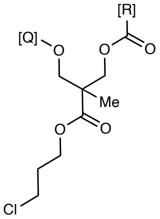

TMCPrCl

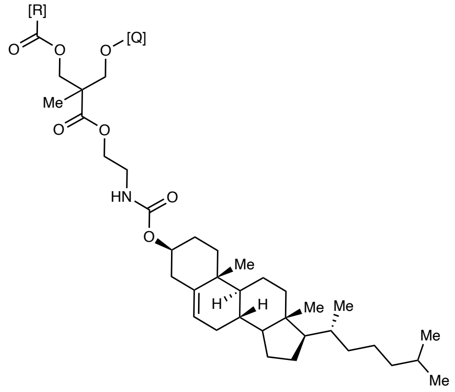

TMCEtNHCOChol

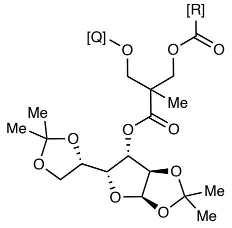

TMCiPPGlucC4

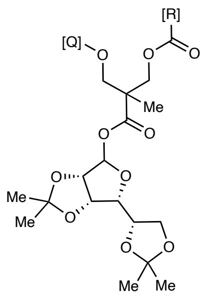

TMCiPPManC1

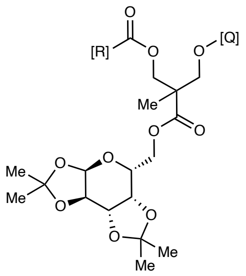

TMCiPPGlacC6

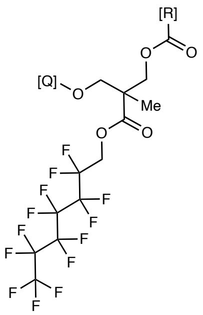

TMCPFPent

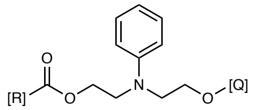

PhDOA

Supplementary Fig. 27. Structures for fragments in Fig. 9b.

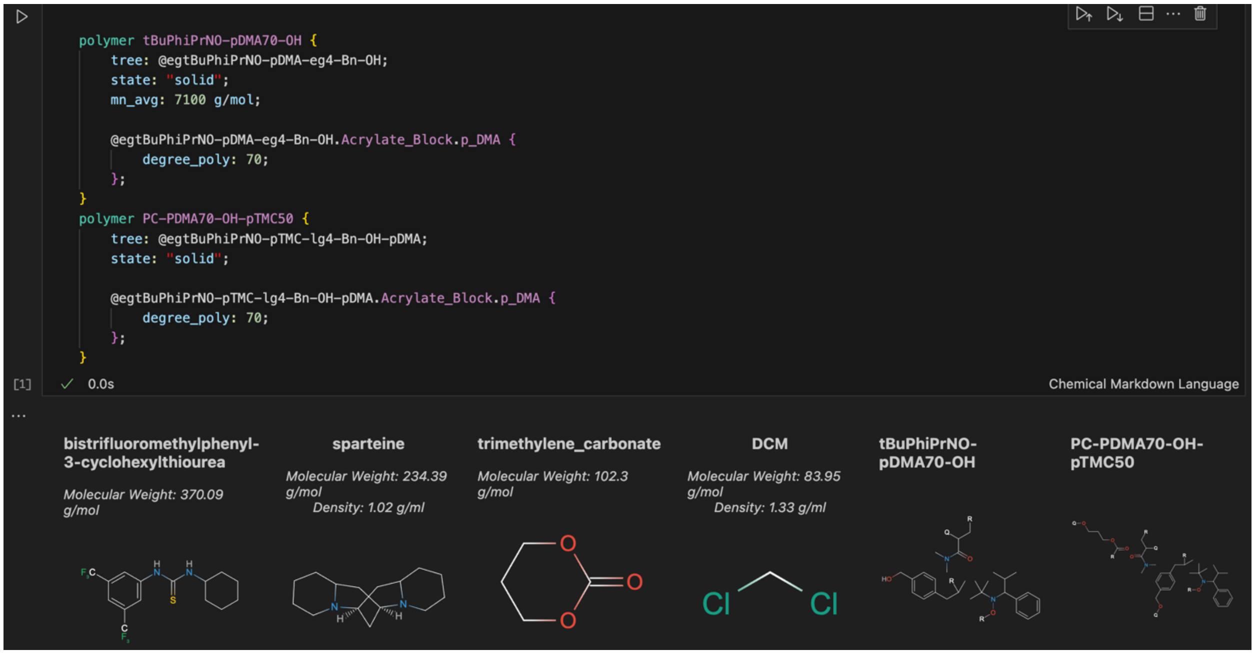

Supplementary Fig. 28. Example of CMDL polymer definitions for macroinitiator (tBuPhPhiPrNO-pDMA70-OH) and block-copolymer product (PC-PDMA70-OH-pTMC50) containing 6c. Connectivity between graph node components tBuPhiPrNO, DMA, 4BnOH, and TMC (Supplementary Figure 27) can be seen in Fig. 9b. Each polymer definition references an imported polymer graph in the *tree* property like in Supplementary Figure 11. For additional details on imports in CMDL notebooks see the IBM Materials Notebook GitHub repository and documentation website.

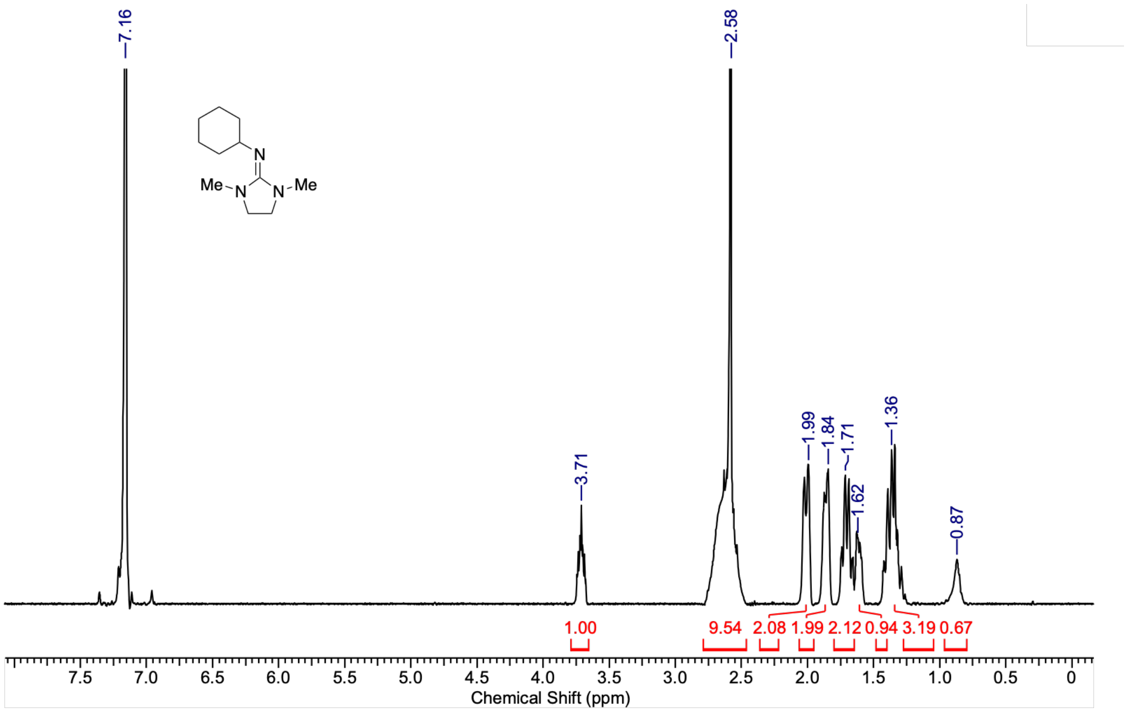

Supplementary Fig. 29. ^{1}H NMR ($CDCl_3$) spectrum of 5c.

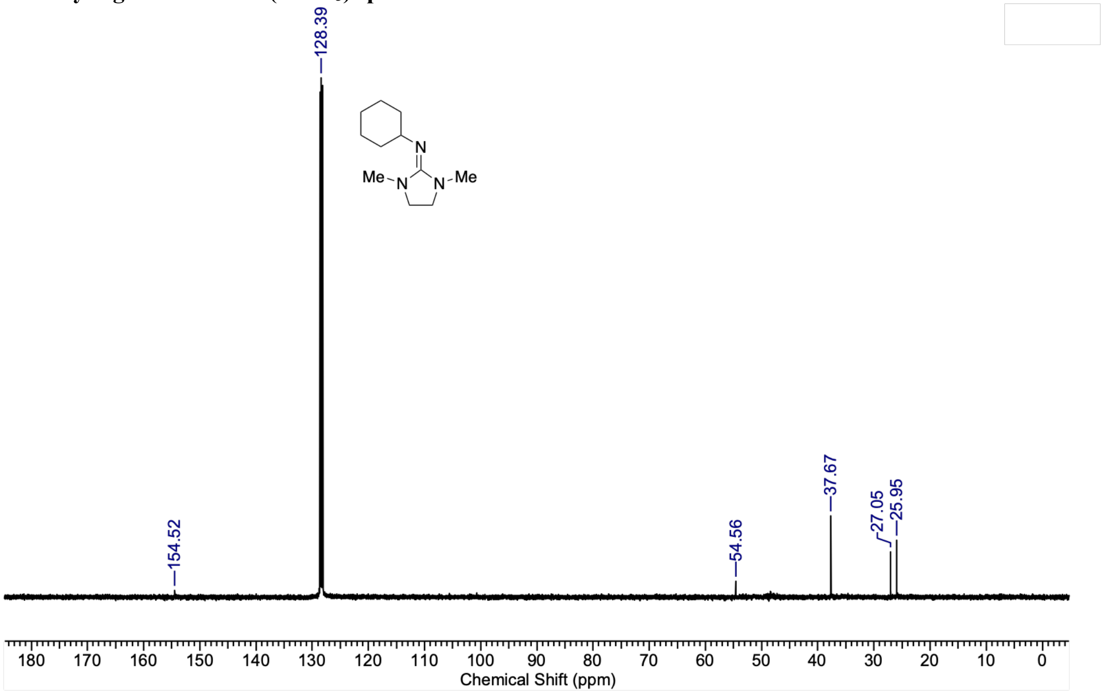

Supplementary Fig. 30. ^{13}C NMR ($CDCl_3$) spectrum of 5c.

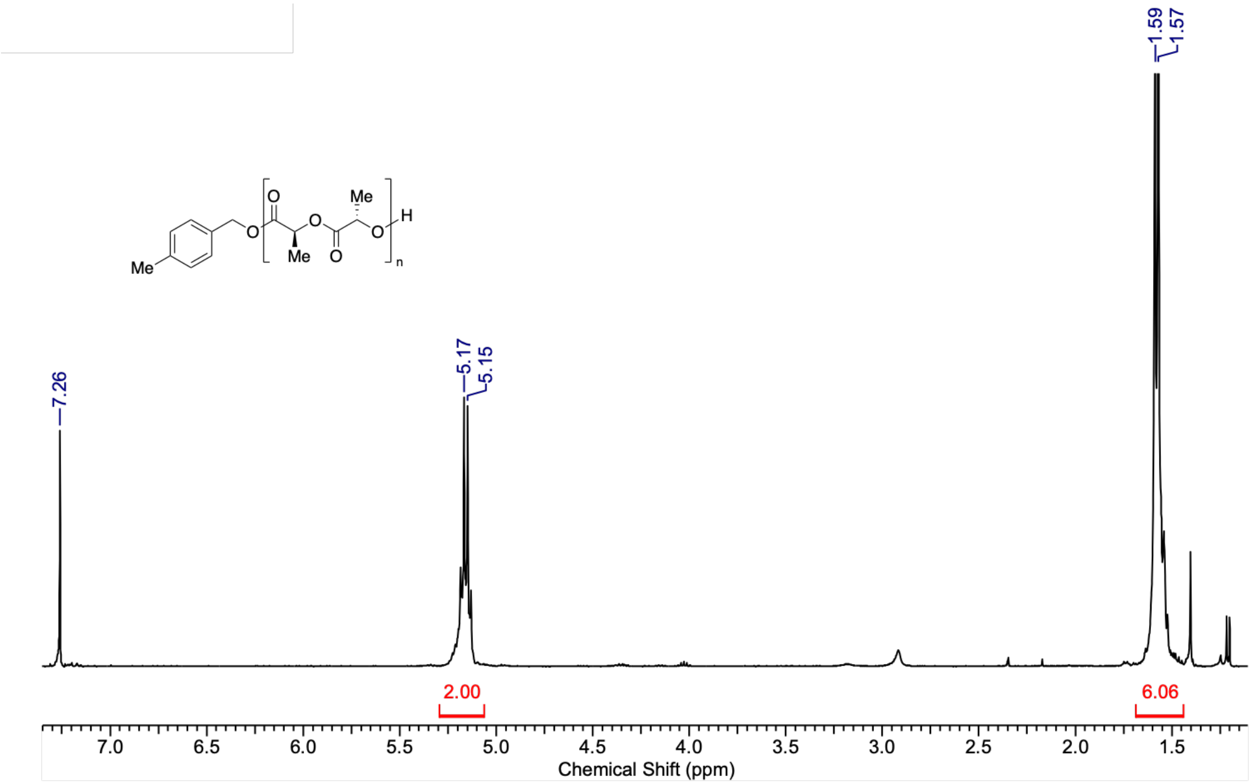

Supplementary Fig. 31. ^{1}H NMR ($CDCl_3$) polymer of 2a (Table 1, entry 3).

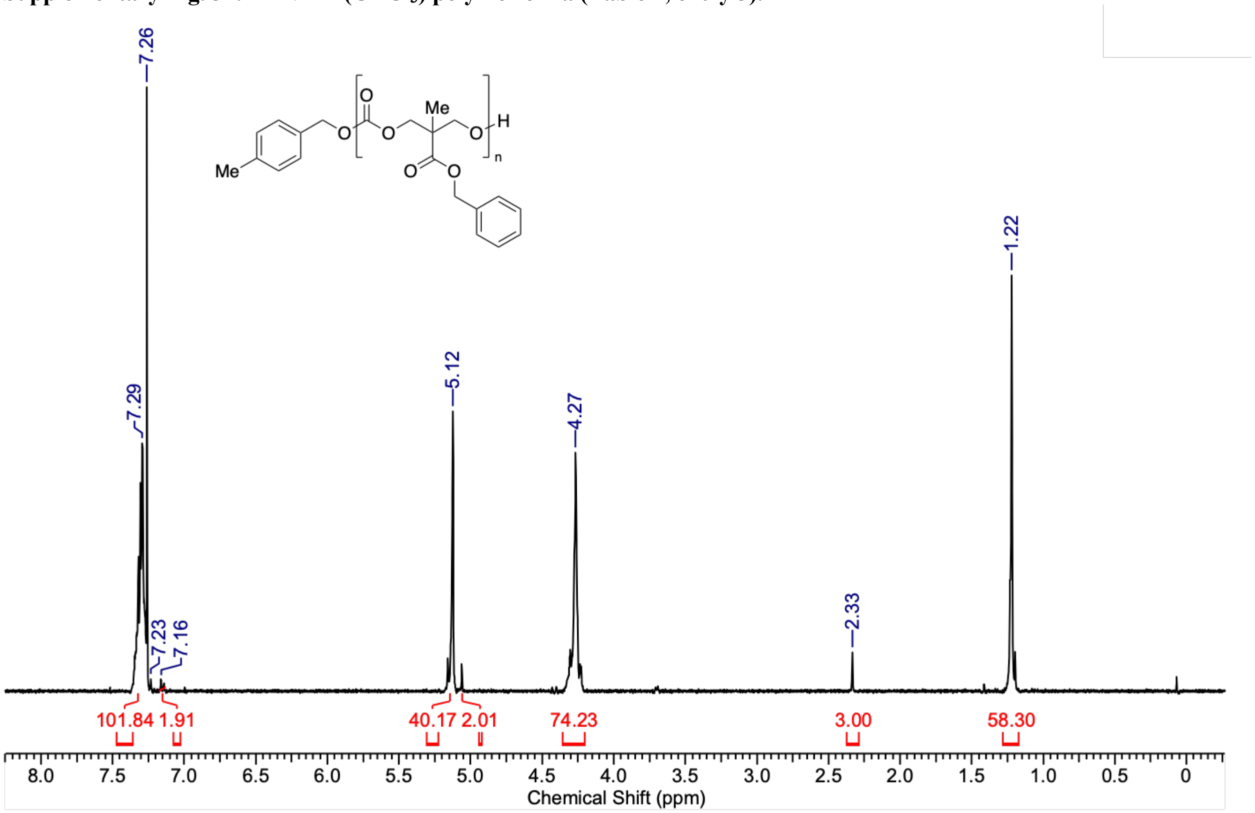

Supplementary Fig. 32. ^{1}H NMR ($CDCl_3$) of polymer of 2d (Table 1, entry 7).

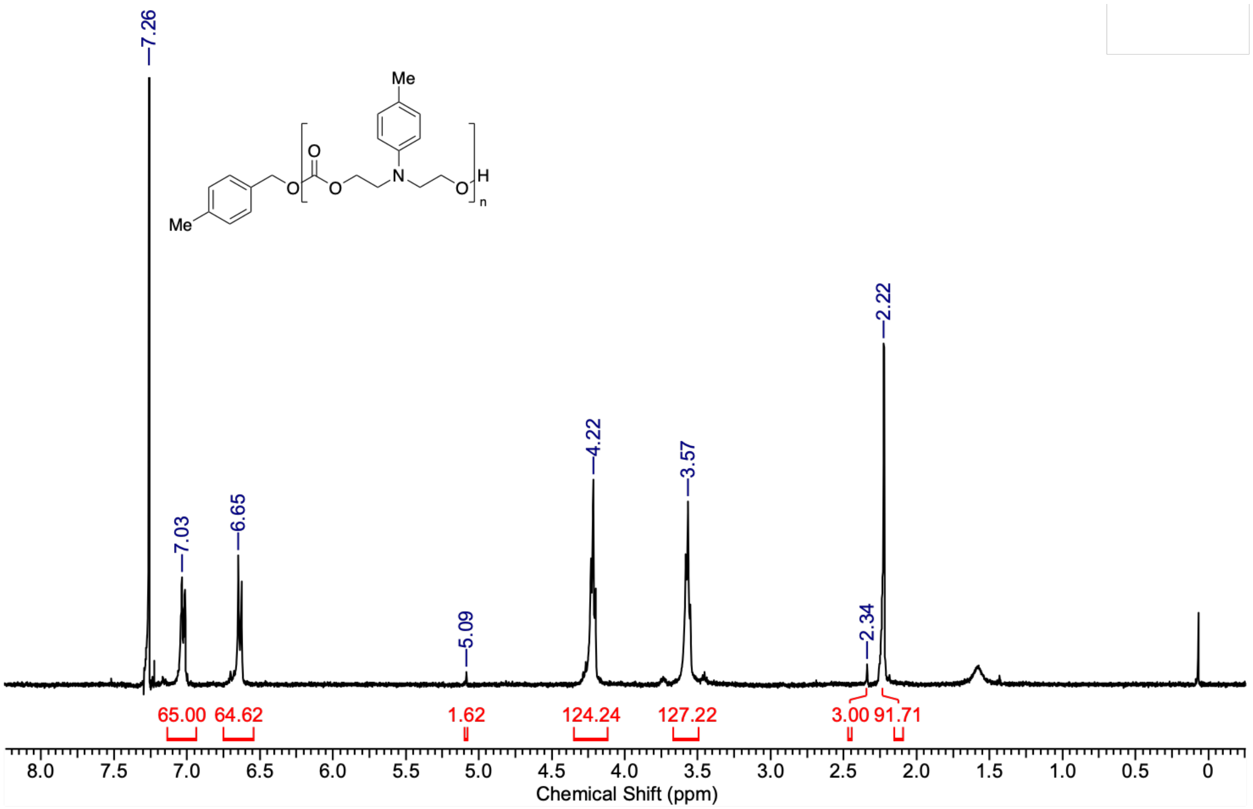

Supplementary Fig. 33. ^{1}H NMR ($CDCl_3$) polymer of 2f (Table 1, entry 9).

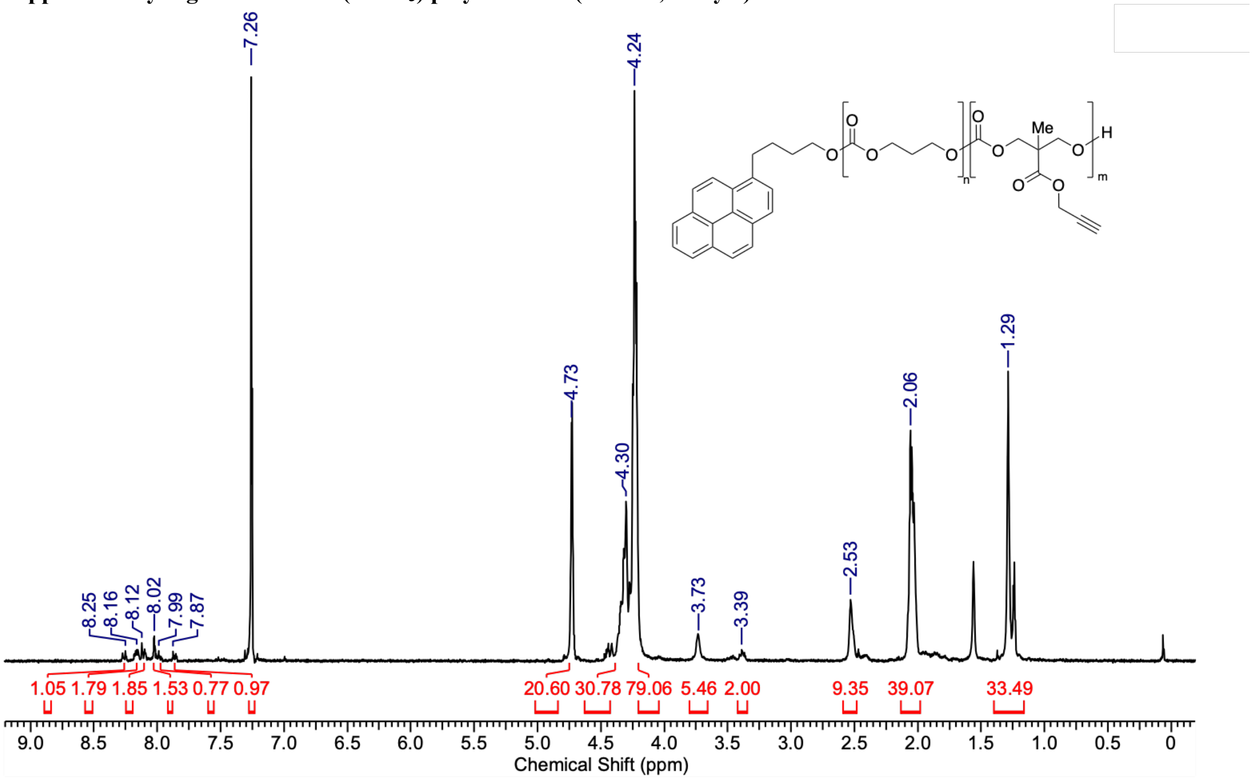

Supplementary Fig. 34. ^{1}H NMR ($CDCl_3$) spectrum of 6e.

Supplementary References

1. Tan, E. W. P. *et al.* Overcoming Barriers in Polycarbonate Synthesis: A Streamlined Approach for the Synthesis of Cyclic Carbonate Monomers. *Macromolecules* **54**, 1767–1774 (2021).

2. Hedrick, J. L., Piunova, V., Park, N. H., Erdmann, T. & Arrechea, P. L. Simple and Efficient Synthesis of Functionalized Cyclic Carbonate Monomers Using Carbon Dioxide. *ACS Macro Lett.* **11**, 368–375 (2022).

3. Dinh, A. N. *et al.* Photocatalytic Oxidative C–H Thiolation: Synthesis of Benzothiazoles and Sulfenylated Indoles. *Synlett* **30**, 1648–1655 (2019).

4. Silva, L., Affeldt, R. F. & Lüdtke, D. S. Synthesis of Glycosyl Amides Using Selenocarboxylates as Traceless Reagents for Amide Bond Formation. *J. Org. Chem.* **81**, 5464–5473 (2016).

5. Li, M. *et al.* Synergetic Organocatalysis for Eliminating Epimerization in Ring-Opening Polymerizations Enables Synthesis of Stereoregular Isotactic Polyester. *J. Am. Chem. Soc.* **141**, 281–289 (2019).

6. Tsuchiya, Y., Kumamoto, T. & Ishikawa, T. Guanidines as a Nitrogen Source for Direct Conversion of Epoxides to Aziridines. *J. Org. Chem.* **69**, 8504–8505 (2004).

7. Pratt, R. C., Nederberg, F., Waymouth, R. M. & Hedrick, J. L. Tagging alcohols with cyclic carbonate: a versatile equivalent of (meth)acrylate for ring-opening polymerization. *Chem Commun* 114–116 (2008) doi:10.1039/B713925J.
